# Individual and Sex Differences in Behavior and Cortical Excitability in the Rat Valproate Model of Autism

**DOI:** 10.3390/cells15171617

**Published:** 2026-09-05

**Authors:** Viktor Kelemen, Zsuzsanna Szeredi-Faragó, Júlia Puskás, Sándor Borbély, Norbert Bencsik, Attila Szűcs, Petra Varró

**Affiliations:** 1 Department of Physiology and Neurobiology, Institute of Biology, Eötvös Loránd University, Pázmány Péter sétány 1/c, 1117 Budapest, Hungaryborbely.sandor@koki.hun-ren.hu (S.B.); szucs.attila3@semmelweis.hu (A.S.); varro.petra@ttk.elte.hu (P.V.); 2 Neuronal Network and Behavior Research Group, HUN-REN Institute of Experimental Medicine, Szigony u. 43, 1083 Budapest, Hungary; 3 Hungarian Centre of Excellence for Molecular Medicine–Semmelweis University (HCEMM-SU), Neurobiology and Neurodegenerative Diseases Research Group, Institute of Clinical Pathophysiology, Semmelweis University, Tűzoltó u. 37-47, 1094 Budapest, Hungary; 4 Human Neuron Physiology and Therapy Core Group, Hungarian Centre of Excellence for Molecular Medicine, Budapesti út 9, 6728 Szeged, Hungary

**Keywords:** autism spectrum disorder, sensorimotor development, social behavior, brain slice, electrophysiology, intrinsic optical signal, entorhinal cortex, prefrontal cortex, excitability

## Abstract

**Highlights:**

**What are the main findings?**

- Prenatal valproate treatment leads to increased excitability of the mPFC in male rats, and the strength of this effect is consistent with the degree of congenital malformations and behavioral deficits.
- In female offspring, significant and complex alterations develop in mPFC functions, despite lesser behavioral deficits compared to males.

**What are the implications of the main findings?**

- In male offspring, malformations appear to be a good predictor of alterations in brain network functions.
- The relationship between cortical network alterations and behavior warrants further studies in female rats prenatally treated with valproate.

**Abstract:**

The rodent prenatal valproate (VPA) treatment is a widely used animal model of idiopathic autism spectrum disorder (ASD). However, the presence of autistic-like symptoms is highly variable in treated offspring. The disruption of the excitation–inhibition balance of certain brain areas has been proposed as a main feature in both human ASD and the VPA model. The current study presents a detailed analysis of neural development, diverse behaviors, and neocortical excitability in a high number of individually identified VPA-treated rat offspring of both sexes. Neocortical excitability was assessed using electrophysiological and intrinsic optical imaging methods. Prenatal VPA treatment caused a delay in early postnatal sensorimotor development in rat pups of both sexes. Behavioral effects were associated with congenital morphological alterations (e.g., tail kink), as shown by stratification by principal component analysis and correlation analysis. Social deficits were evident only in the VPA-treated male offspring, while the females appear to be resistant to this effect. Prenatal VPA treatment was associated with sex- and region-dependent alterations in the excitability and seizure susceptibility of entorhinal and prefrontal cortical slices. Cortical excitability measures showed partial correlation with morphological and behavioral parameters. Thus, the current study further supports the validity of the rodent prenatal VPA model as a model of ASD for both sexes, but the variable degree of affectedness should be taken into account. Congenital malformation severity, including tail kink, may serve as a readily observable marker for subsequent physiological alterations, particularly in males.

## 1. Introduction

Autism spectrum disorder (ASD) is a neurodevelopmental disorder characterized by two core symptom groups, namely social deficits and restrictive, repetitive behaviors, according to the Diagnostic and Statistical Manual of Mental Disorders [1]. Both genetic background and environmental factors to which the developing fetus is exposed (e.g., maternal immune activation or certain chemicals) may influence the manifestation of the disorder [2]. A total of 5–15% of ASD cases have a well-defined monogenic origin (so-called syndromic autism), but the rest are of unknown cause, so-called idiopathic autism cases. The severity and the symptoms are highly variable [3]. Overall, the development of the disorder is not sufficiently well understood, and no specific therapy targeting the core symptoms exists to date. This makes further study of preclinical animal models a valuable source of information.

Several types of alterations in the brain have been described in ASD so far. Alterations in developmental timeline, structure, and connectivity in several brain areas, including the cerebellum, neocortical, and limbic structures, have been reported [4,5]. These alterations may be explained by altered neuronal migration, survival, or synapse formation. Among the multitude of risk genes that have been linked to ASD, there are genes regulating chromatin remodeling, transcription, protein degradation, and genes coding proteins participating directly in synaptic transmission (cell adhesion molecules, neurotransmitter receptors, and scaffold proteins). Altered connectivity leads to altered synaptic homeostasis [6], and more specifically, the balance of excitatory and inhibitory processes may be disrupted.

The excitation–inhibition imbalance hypothesis of autism [7] postulates a predominance of excitatory processes in key brain areas, which may be the result of either excitatory or inhibitory dysfunctions or homeostatic plasticity processes [8]. Dysfunctions linked to neocortical interneurons have been evidenced in both human autism and animal models [9]. ASD displays high comorbidity with epilepsy, a disease in which the predominance of excitatory processes leads to spontaneous seizures. The possible genetic background of the two conditions overlaps, and the dysfunction of inhibition appears to be a common feature in both cases [10].

The rodent prenatal valproate (VPA) model is one of the most widely used animal models of idiopathic ASD. VPA is an antiepileptic, mood-stabilizing drug that increases the risk of ASD in human offspring if taken during the first trimester of pregnancy [11]. This side effect has been exploited to generate ASD models using diverse experimental animals, including zebrafish [12]. If VPA is administered to rodents at a high dose during a critical neurodevelopmental window, it elicits autism-like behavior in rodents, too. The model has been extensively analyzed, and many aspects also present in human cases have been evidenced; thus, it is considered an ASD model with relatively high validity [13,14]. VPA is a histone deacetylase inhibitor, thus influencing gene transcription, and this seems to be the main mechanism by which it induces autism-like behavior in rodents when applied prenatally. The VPA rodent model also displays the excitation–inhibition imbalance characteristic of autism, but symptom severity in treated pups is considerably variable compared to monogenic ASD models [15].

Behavioral experiments applied in animal studies of ASD often include early postnatal tests on sensorimotor development, because the delay of sensorimotor maturation has also been described in autistic children. Such tests typically include the negative geotaxis reflex, surface righting reflex, visual placing reflex, and auditory startle reflex [16]. Other tests aiming at the core symptoms of ASD are carried out in a later period of life: repetitive behavior is usually tested with marble burying and grooming tests [17]; autism model animals perform these behavioral elements more frequently. For testing social deficits, the three-chamber social interaction test is most widely used (alternatives are the study of social play with direct contact or quantification of ultrasonic vocalization in pups) [18].

An interesting phenomenon about ASD is the strong gender bias of its incidence. Historically, a 4–5:1 male:female ratio has been reported for ASD [13]. For this reason, in most animal studies applying the VPA model, only male offspring have been examined [16,19,20]. However, newer studies about ASD raise questions about the male preponderance, explaining the bias by different manifestations of the disorder in females, concealment of symptoms, and underdiagnosis [21,22,23]. In rodent VPA model studies distinguishing between female and male pups, most studies report that males display more profound social deficits, but other behaviors and brain morphology are altered in females, too [24,25].

Several brain areas show alterations in ASD, as mentioned previously. The medial prefrontal cortex (mPFC) is considered a key regulator for social cognition and extensively studied in the context of ASD. Indeed, it exhibits altered growth rate, connectivity, and columnar structure in autistic patients [5]. The entorhinal cortex is not as thoroughly studied in the context of ASD. It is an intermediate cortical region connecting the hippocampus to associative neocortical areas, which, due to its circuitry and connections, is one of the key seizure-trigger zones in temporal lobe epilepsy [26]. As excitation–inhibition imbalance is a common feature in ASD and epilepsy, it is well justified to investigate ASD-related alterations in entorhinal cortical excitability.

The aim of the current study was to analyze the outcome of prenatal VPA treatment in a large number of individually followed rat pups of both sexes, to address eventual sex differences and differences in symptom severity. An extensive battery of behavioral tests was used to examine early nervous system development and autistic-like behaviors. Based on the results of these multiple behavioral tests, a principal component analysis (PCA) was carried out to gain a visual representation of the distribution of the characteristics of individual control and VPA-treated pups. As the VPA-treated rats showed high variability, they were further stratified as weakly or strongly affected by the treatment based on how much their results differed from the control population. This subgrouping was utilized for the subsequent brain slice studies. The excitability of prefrontal and entorhinal cortical brain slices was studied on the network level using electrophysiological and intrinsic optical recording techniques. To address subsequent developmental changes or compensatory mechanisms shaping cortical excitability, two age groups were studied. As an attempt to mathematically quantify the association between morphological, behavioral, and cortical excitability parameters, correlation analysis of the data was also performed.

## 2. Materials and Methods

The design of this study was in accordance with the European Union directive 2010/63/EU, and the animal study protocol was approved by the Eötvös Loránd University Animal Care Committee and by the Hungarian National Animal Health Care Authority (license no. PE/EA/772-7/2020). All possible efforts were made to decrease the animal number and suffering.

### 2.1. Animals and Valproate Treatment

Adult male and female Wistar rats (Toxi-Coop Ltd., Budapest, Hungary) were used for breeding. We used the pups for the behavioral and electrophysiological tests. The rats were kept at a controlled temperature (22 ± 2 °C) under a constant 12 h light/dark cycle. Standard pellet food (Toxi-Coop Ltd., Budapest, Hungary) and tap water were available ad libitum.

**Figure 1:**
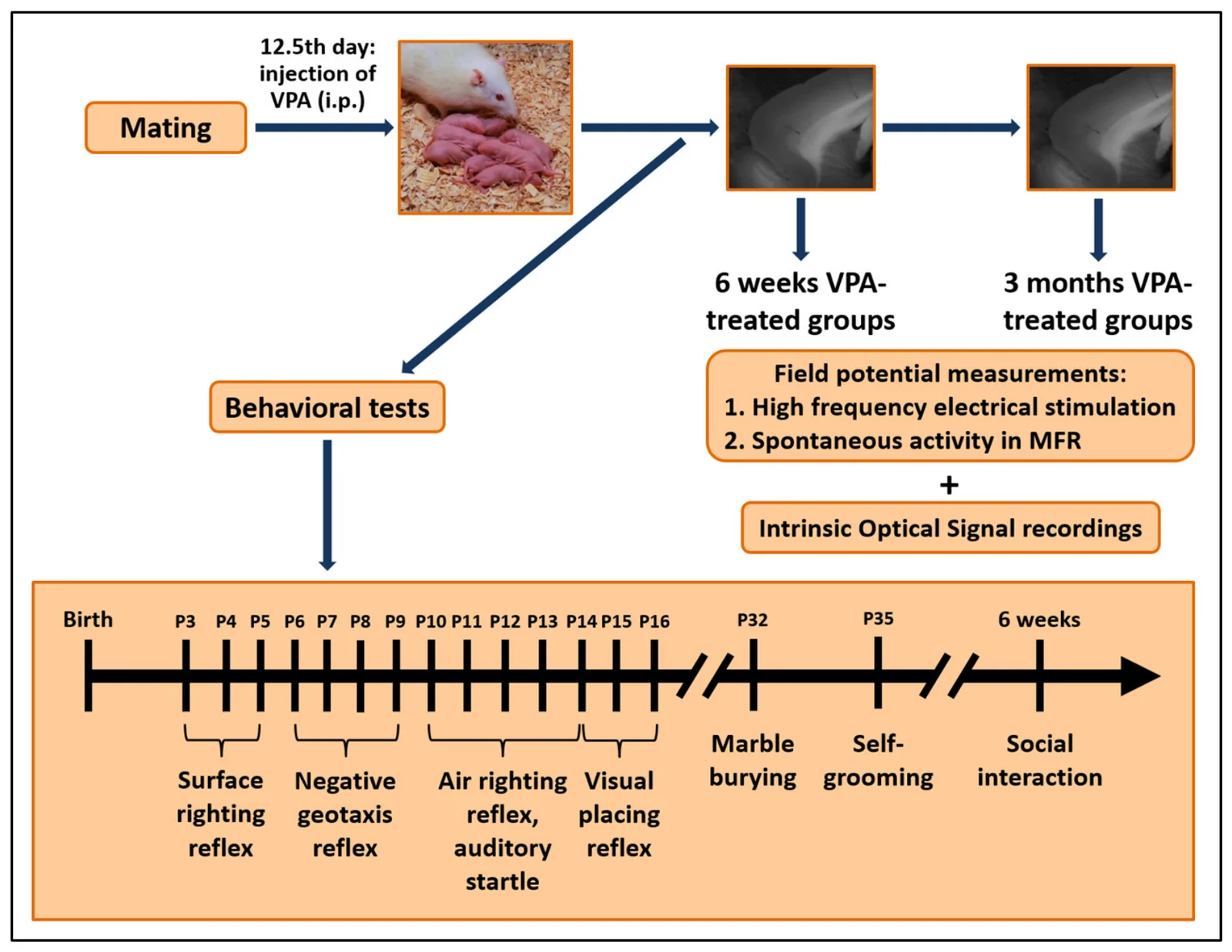
A timeline of the experimental protocols. The pregnant Wistar rats received an i.p. injection of VPA on gestation day 12.5 (dose 500 mg/bw kg). The pups were tested for various motor functions and behaviors from postnatal day 3 (P3) to week 6. Electrophysiological studies were performed in two age groups: 6 weeks and 3 months. Brain slices were prepared from the offspring for field potential measurements and parallel detection of intrinsic optical signals. The excitability changes were tested with spontaneous bursts evoked by Mg^2+^-free solution (MFR) and afterdischarges (ADs) evoked by brief bursts of high-frequency electrical stimulation.

The pregnancy of rat dams was confirmed by the presence of a vaginal plug or by the presence of sperm in the vaginal smear. The rat dams were treated with a single intraperitoneal injection of sodium valproate (Sigma-Aldrich Ltd., Budapest, Hungary) dissolved in 0.9% saline (the concentration of the solution was 150 mg/mL, dosage: 500 mg/bw kg) on embryonic day 12.5, while other dams were injected with saline at the same time of gestation (control group) [13,27,28]. Cages containing treated and control rats were kept in the same animal facility room. The timeline of experiments is summarized in Figure 1.

### 2.2. Behavioral Testing and Data Analysis

Pups of both sexes were used for the experiments. They were observed for congenital malformations, and a **deformity score** was assigned to them accordingly. A simple tail kink or one more or one less toe on one limb was scored as one point; a double tail kink was scored as two points; and a triple tail kink or the absence of a limb was scored as three points. Each malformation was noted, and the scores were summed to obtain the deformity score. If no malformations were observed on the pup, the deformity score was 0.

The pups were tested for various motor functions and behaviors from postnatal day 3 (P3) to week 6.

**Surface righting reflex.** The pups (from P3 to P5) were carefully placed on a flat surface in a supine position. The individuals’ test was deemed successful if they flip to their feet in 4 s. If the pups could not perform it until P5, the test was carried out on each day until a successful test was performed.

**Negative geotaxis reflex.** The performance of the pups during the negative geotaxis test reflects the vestibular function, motor development, and activity. The pups (from P6 to P9) were tested on a 30° inclined surface, where they were placed head down, and a 180° turn was performed. If the pups could not perform it until P9, the test was carried out on each day until a successful test was performed.

**Air righting reflex.** The animals (from P10 to P14) were held in a supine position 30 cm over thickened bedding, then dropped from this height. This was repeated 3 times, and how many feet they landed on was registered. The test was considered successful if the pup landed on 3 or 4 legs at least 2 times out of 3.

**Auditory startle response.** The rats (from P10 to P14) were surprised by a loud noise, a sudden clap by the experimenter. If the animal exhibited the startle response, meaning its whole body flinches, the test was deemed successful.

**Visual placing reflex.** From P13, the rats’ eyes were observed as to whether they were opening. The visual placing test is used to ensure that beside the eyes being opened, the visual cues are properly processed. For the test, the pups with eyes already opened (from P13 to P16) were lifted by their tail and the experimenter lowered them slowly towards the surface. If the animal extended the forelimbs towards the surface during the test, it succeeded at the test.

**Marble burying.** This test was used to portray obsessive–compulsive behavior and anxiety in the rats. The animals (P32) were placed on thickened bedding, where 16 marbles were aligned. After 20 min the number of marbles buried under the bedding was registered.

**Self-grooming.** Repetitive behaviors were tested in an open field apparatus. The pups (P35) were placed into an arena for 15 min, where the walls and the floor are black, but the experimenter and the camera can have a good sight from above. Whenever they exhibited grooming behavior, defined as the double-handed forearm strokes focusing on the head area, the experimenter recorded the length of the behavior.

**Social interaction test.** The social behavior of the rats was examined using a three-chamber test. The animals were placed into a black rectangular box, which is divided into 3 equal parts, connected by openings. The middle section does not contain any objects, while there is a cage in both other sections. The test rat was placed into the middle part. During the first 10 min, the cages were empty, and the animal could freely explore the arena. After that, another non-familiar rat, similar in age and same sex to the test rat, was placed under one of the cages for 10 min. The experimenter evaluated how much time the test rat spent in each section of the box. The social preference index is calculated from the time spent with another rat vs. the time spent with the inanimate object (cage) using the following formula:

I_SP_ = (T_S_ − T_NS_)/(T_S_ + T_NS_),

where I_SP_ is the social preference index, T_S_ is the interaction time with the social stimulus, and T_NS_ is the interaction time with the non-social stimulus [29].

**PCA.** Autism is a spectrum disorder, meaning that it can manifest very differently in different individuals. The rodent VPA model seems to reproduce this aspect of the disorder, as alterations manifest to a different degree in each treated animal. Taking into account the results of the behavioral tests and the severity of the malformations, the treated animals were divided into two groups using principal component analysis. The statistical analysis was performed using Origin software (Origin 2019b, OriginLab Corporation, Northampton, MA, USA). The scores of the individual rats were plotted against the first two principal components. Subgrouping of the VPA-treated rats was performed based on z-score values: the distance from the centroid of the point cloud of the control rats was calculated: if z < 1.75, the individual rat was classified into the “VPA weak” subgroup, if z > 1.75, then it was classified into the “VPA strong” subgroup.

### 2.3. Brain Slice Preparation

Experiments on brain slices were carried out at 6 weeks and 3 months of age. Anesthesia was performed with i.p. administration of chloral hydrate (solution: 140 mg/mL, dosage: 350 mg/bw kg). Then, the rats were decapitated and the whole brain was removed and put in 4 °C choline chloride-based cutting solution (composition in mM: 92 choline chloride; 2.5 KCl; 1.2 NaH_2_PO_4_; 30 NaHCO_3_; 20 HEPES; 25 glucose; 5 sodium ascorbate; 2 thiourea; 3 sodium pyruvate; 10 MgSO_4_·7H_2_O; 0.5 CaCl_2_·2H_2_O). Four hundred micrometer thick horizontal (for registering field potentials in the entorhinal cortex) or sagittal brain slices (for registering field potentials in the medial prefrontal cortex) were cut with a vibratome (Electron Microscopy Sciences, Hatfield, PA, USA). The slices were incubated in carbogenated artificial cerebrospinal fluid (ACSF) for at least 1 h at room temperature. The precise composition of ACSF was (in mM): 126 NaCl; 26 NaHCO_3_; 1.8 KCl; 1.25 KH_2_PO_4_; 2.4 CaCl_2_·2H_2_O; 1.3 MgSO_4_·7H_2_O; and 10 glucose (pH 7.2–7.4).

### 2.4. Electrophysiological Recording and Data Analysis

For recording, the slices were transferred to a Haas-type interface chamber (Experimetria Ltd., Budapest, Hungary) and kept at 33 ± 1 °C, while continually perfused with carbogenated ACSF. A bipolar tungsten stimulation electrode was placed at the border between the gray and white matter (square voltage pulses of 100 µs width, BioStim, Supertech Ltd., Pécs, Hungary). First, the viability of the slices was tested at a low voltage stimulation (2.4–6 V), and then the voltage value was continuously increased every 2 min until ADs were evoked. This voltage value was called the AD threshold.

**Figure 2:**
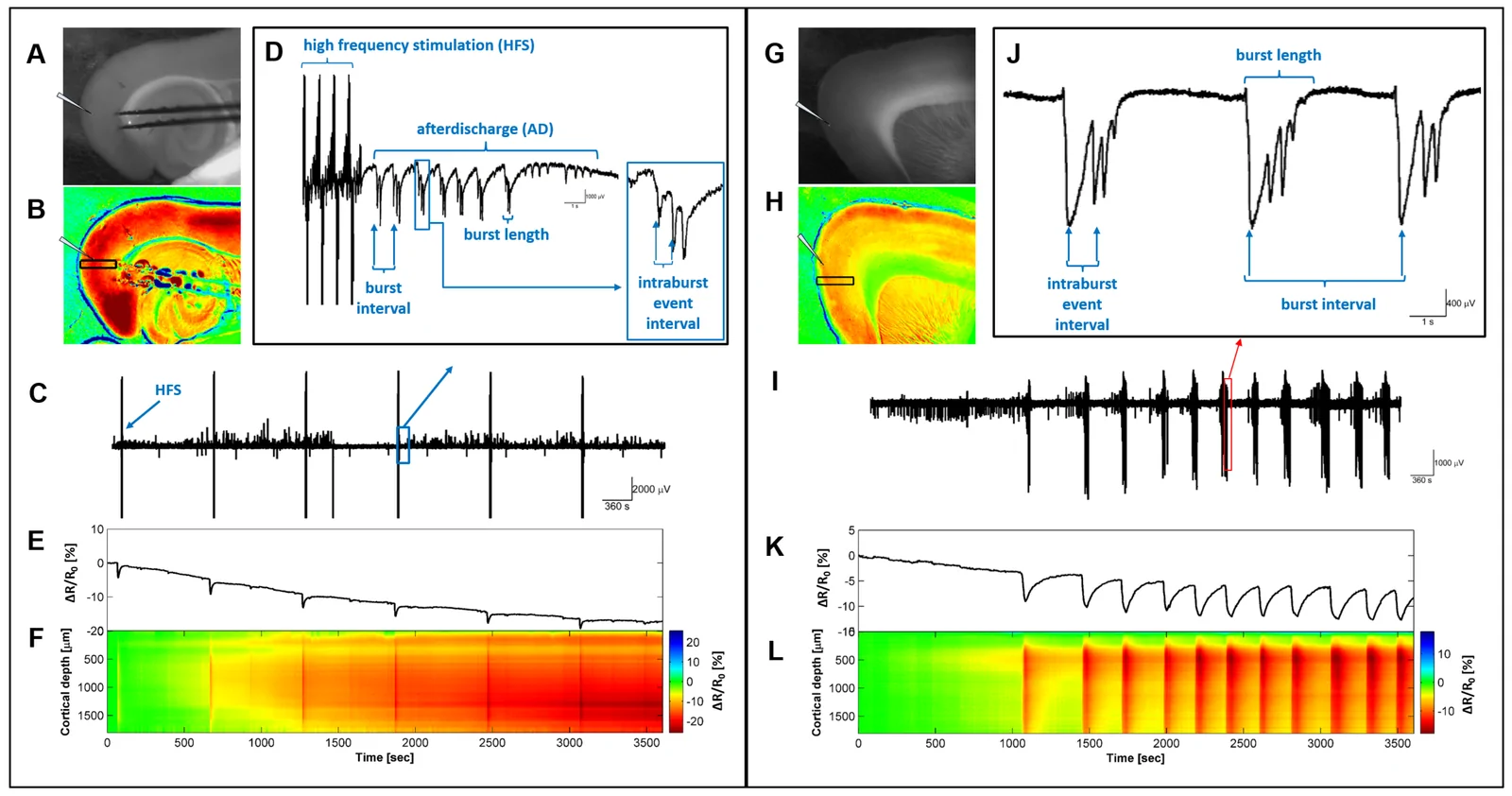
An overview of a single field potential and intrinsic optical signal measurement. (**Left panel**): The afterdischarges evoked by high-frequency stimulation. (**A**) The positioning of the electrodes for registering field potentials in the entorhinal cortex: stimulation at the border of the gray and white matter and recording (glass electrode marked with light blue triangle) in layer II/III. (**B**) The relative change in light reflectance from an entorhinal slice with ROI (black rectangle) after high-frequency stimulation (HFS). (**C**) The whole (3600 s) recording of electrical signals: HFS was delivered every 10 min, a total of 6 times during the measurement. (**D**) Single HFS, the induced AD, and the measured parameters. (**E**) The averaged relative reflectance change in the analyzed area (black rectangle in (**B**)) plotted against time. (**F**) The relative reflectance change in every pixel of the analyzed area. The vertical bands correlate with the occurrence of HFSs followed by ADs. (**Right panel**): The spontaneous activity in the MFR. (**G**) Positioning of the electrode for registering field potentials in the prefrontal cortex: the recording electrode (glass electrode marked with light blue triangle) in layer II/III. (**H**) The relative change in light reflectance from a prefrontal slice with ROI (black rectangle) after spontaneous activity in the MFR. (**I**) The whole (3600 s) recording of electrical signals. (**J**) The measured electrophysiological parameters. (**K**) The averaged relative reflectance change in the analyzed area (black rectangle in (**H**)) plotted against time. (**L**) The relative reflectance change in every pixel of the analyzed area. The vertical bands correlate with the occurrence of high-amplitude, long bursts.

Following the initial assessment of responses, a 60 min recording was started. ADs were evoked by 5 s long 50 Hz stimulation with a voltage corresponding to the AD threshold. Field response for this high-frequency stimulation (HFS) was recorded with an extracellular glass microelectrode filled with 1 M NaCl (5–15 MΩ), positioned in layer II/III of the entorhinal and prefrontal cortex (Figure 2A). ADs were elicited every 10 min during the recording.

In another set of slices, MFR was used to elicit spontaneous epileptiform activity. The viability of the slices was checked by evoking a field potential with electrical stimulation, as described before. Then, only the glass recording electrode was left in place. After switching the perfusion solution from ACSF to MFR, the latency time of the first ictal-like activity was noted. Then, a 60 min recording was started.

Electrical signals were amplified (1000×, Axoclamp 2B, Axon Instruments Inc., Union City, CA, USA), filtered (0.6–500 Hz, Supertech Ltd., Pécs, Hungary), and stored (sampling rate 20 kHz) using custom-written MATLAB-based software (version by S. Borbély), together with the intrinsic optical signal.

AD waveforms were analyzed manually using the MathWorks MATLAB-based FPanalyzer software (FPanalyzer v. 2015 by S. Borbély) to determine the following parameters: total length of ADs, length of each burst, interval between bursts (used to calculate burst frequency), and intraburst event interval (used to calculate intraburst frequency) (Figure 2D). The parameters were calculated as an average of the 6 AD series evoked during the 60 min recording.

Field potentials obtained during MFR perfusion were analyzed by the NeuroExpress software (NeuroExpress v. 21.9.07, by A. Szűcs), using automated peak detection. Burst length, burst interval (used to calculate burst frequency), and intraburst event interval (used to calculate intraburst frequency) were determined (Figure 2J). The evaluation of electrophysiological (and intrinsic optical) signals was performed at 120 s intervals, starting from the time points corresponding to the HFS (1st, 11th, 21st, 31st, 41st, and 51st minutes). The parameters were calculated as an average of the 6 series during the 60 min recording.

### 2.5. Recording of Intrinsic Optical Signals and Data Analysis

In parallel with the electrophysiological recording, the intrinsic optical signal (IOS) of the slice was also recorded, both for the AD and the MFR experiments. The slice was illuminated from above with a voltage-stabilized, fiber light optical device (Dolan-Jenner MI-150, Dolan-Jenner Industries Inc., Boxborough, MA, USA), and the reflected light was recorded with a CCD camera (Qimaging 3.3, Teledyne Technologies Inc., Thousand Oaks, CA, USA) attached to a trinocular stereo microscope (Nikon SMZ800, Nikon Instruments Inc., Melville, NY, USA at 2 fps.

The recordings were analyzed using the MathWorks MATLAB-based IOSAnalysis2 software (IOS_Analysis2 v. 2017 by S. Borbély). The first twenty frames of each picture series were averaged to generate a control image, and it was subtracted from all consecutive frames using the following formula:

∆R= ((R − R_0_)/R_0_) × 100

where ∆R is the relative reflectance change, and R_0_ is the reflectance of the control image, while R is the reflectance of images during afterdischarge or spontaneous activity.

The region of interest (ROI) was defined as a rectangle containing all of the layers of the entorhinal and prefrontal cortex around the recording electrode. The relative reflectance change was calculated in an ROI for every pixel and averaged by rows to visualize the changes in cortical layers. The relative reflectance change data represent alterations that developed during the 60 min recordings.

### 2.6. Statistical Analysis

The sample sizes in the PCA, deformity score, and behavioral tests were as follows: N = 73, 86, and 46 in the case of males, and N = 80, 60, and 47 in the case of females in the control, VPA weak, and VPA strong groups, respectively. The sample sizes of each electrophysiological measurement can be found in the figure captions.

After verifying the normal distribution of the data, two- or three-way ANOVA (with Levene’s test for homogeneity of variances and Tukey’s post hoc test, *p* < 0.05) was performed for the statistical analysis to estimate the significance of differences between different groups (Origin 2019b, OriginLab Corporation, Northampton, MA, USA). Two-way ANOVA was used to analyze the morphological and behavioral results, taking into account the VPA treatment groups and sexes. Three-way ANOVA was used for the analysis of electrophysiological and IOS results, where treatment, sex, and age group were considered. The different colored columns represent the average value in different sexes (the shades of blue for the males and the shades of pink for the females); the striped (VPA weak) and latticed columns (VPA strong) at the different colorings show the VPA treatment. The black points represent the individual measurement points. The error bar indicates the SEM.

A correlation analysis was performed to examine the degree of association between the recorded morphological, behavioral, and cortical excitability parameters (the latter including both electrophysiological and IOS data). The data for individual rats belonging to a certain treatment subgroup (control, VPA weak, or VPA strong), sex, or age group were subjected to pairwise Pearson’s correlation analysis. The results were plotted on heat maps for easy visualization of correlation strength. The significance of the correlation coefficient was tested by Student’s *t*-test (*p* < 0.05) (Origin 2019b, OriginLab Corporation, Northampton, MA, USA).

## 3. Results

### 3.1. VPA Treatment Affects Rat Pups in a Variable Manner Based on Deformity and Behavior

The results of the deformity scoring and the behavioral test battery carried out on the control and VPA-treated pups were analyzed with PCA, with the aim of studying the correlation between different parameters and capturing eventual parameters with a high predictive power concerning VPA treatment. As VPA treatment is known for high variability in the outcome, our goal was also to discern eventual clusters among the treated pups, according to the severity of their affectedness. Due to technical reasons, the results of the brain slice experiments could not be included in this analysis.

The eigenvalues and the percentage of variance linked to each principal component are shown in Table 1. The first two principal components account for only cca. 40% of the variability of the dataset, and several subsequent components account for a relatively high share.

**Figure 3:**
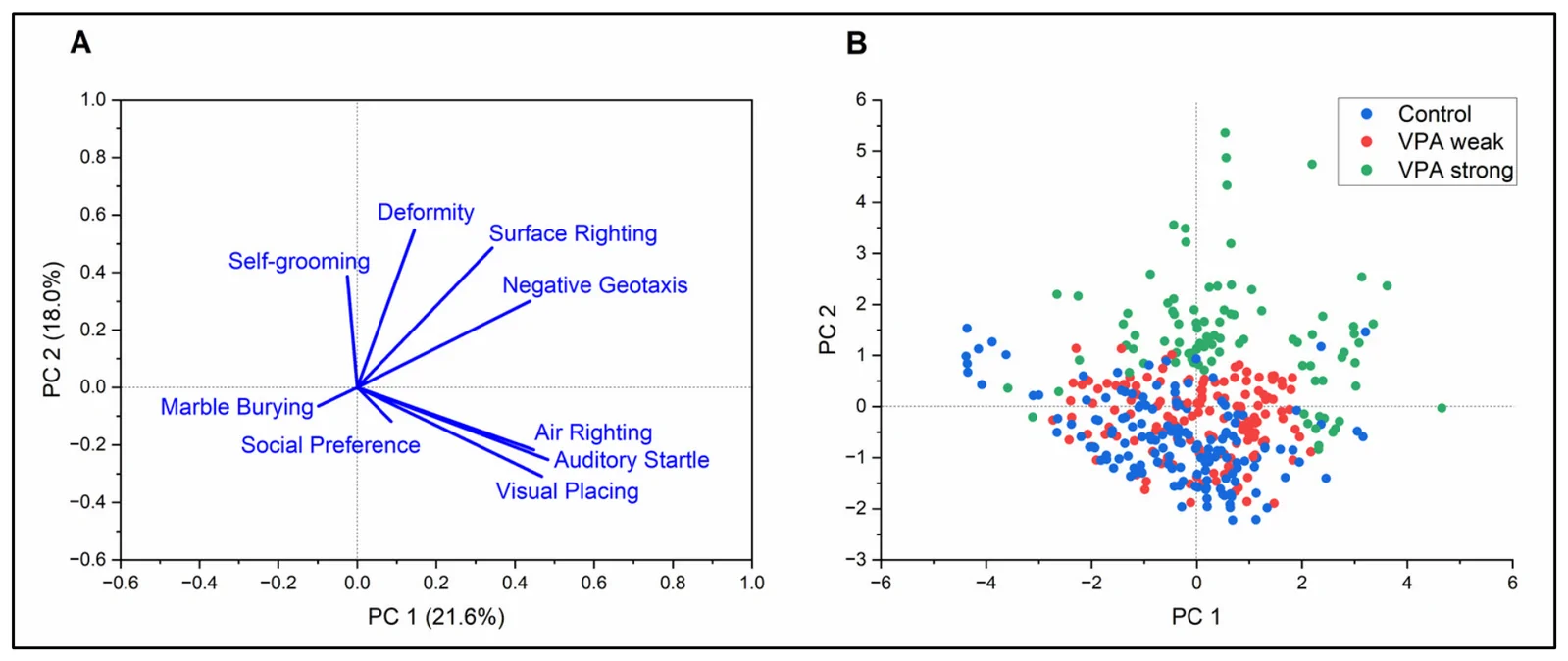
(**A**) The two-dimensional loading plot of the principal component analysis, showing the morphological and behavioral results on PC1 and PC2 as vectors. (**B**) The scores of the individual rats plotted against the first two principal components. Subgrouping of the VPA-treated rats was performed based on z-score values.

The relative importance and correlation of the different variables can be seen on the two-dimensional loading plot of the PCA (Figure 3A). It is conspicuous that the deformity score and the results of sensorimotor reflexes tested during early postnatal development are of high importance, while the contribution of repetitive and social behavioral data is lower. The deformity score correlates with surface righting and negative geotaxis reflex data, which are studied during the first postnatal week. Air righting reflex, auditory startle, and visual placing reflex data form a clear cluster; these behaviors reflect maturation of the nervous system during the second postnatal week.

The distribution of the morphological and behavioral results of the individual rat pups can be seen in Figure 3B. The control and VPA-treated rats form highly overlapping datasets, but while the results from the control rats form a more compact population, the data on the VPA-treated rats display much higher variability: part of the data blends with the control data while part of the data clearly separates from the control group, reflecting higher affectedness by the treatment. Thus, based on the z-score of the individual rats in this visualization mode, the VPA-treated group was further divided into “VPA weak” and “VPA strong” subgroups.

The results of the different behavioral tests and the network excitability data is presented according to this grouping of the animals to allow some correlative analysis of VPA effect on behavior and network excitability (the latter derived from brain slice data).

### 3.2. VPA Affects Sensorimotor Development and Induces Repetitive Behavior and Social Deficits

#### 3.2.1. Sensorimotor Development

**Figure 4:**
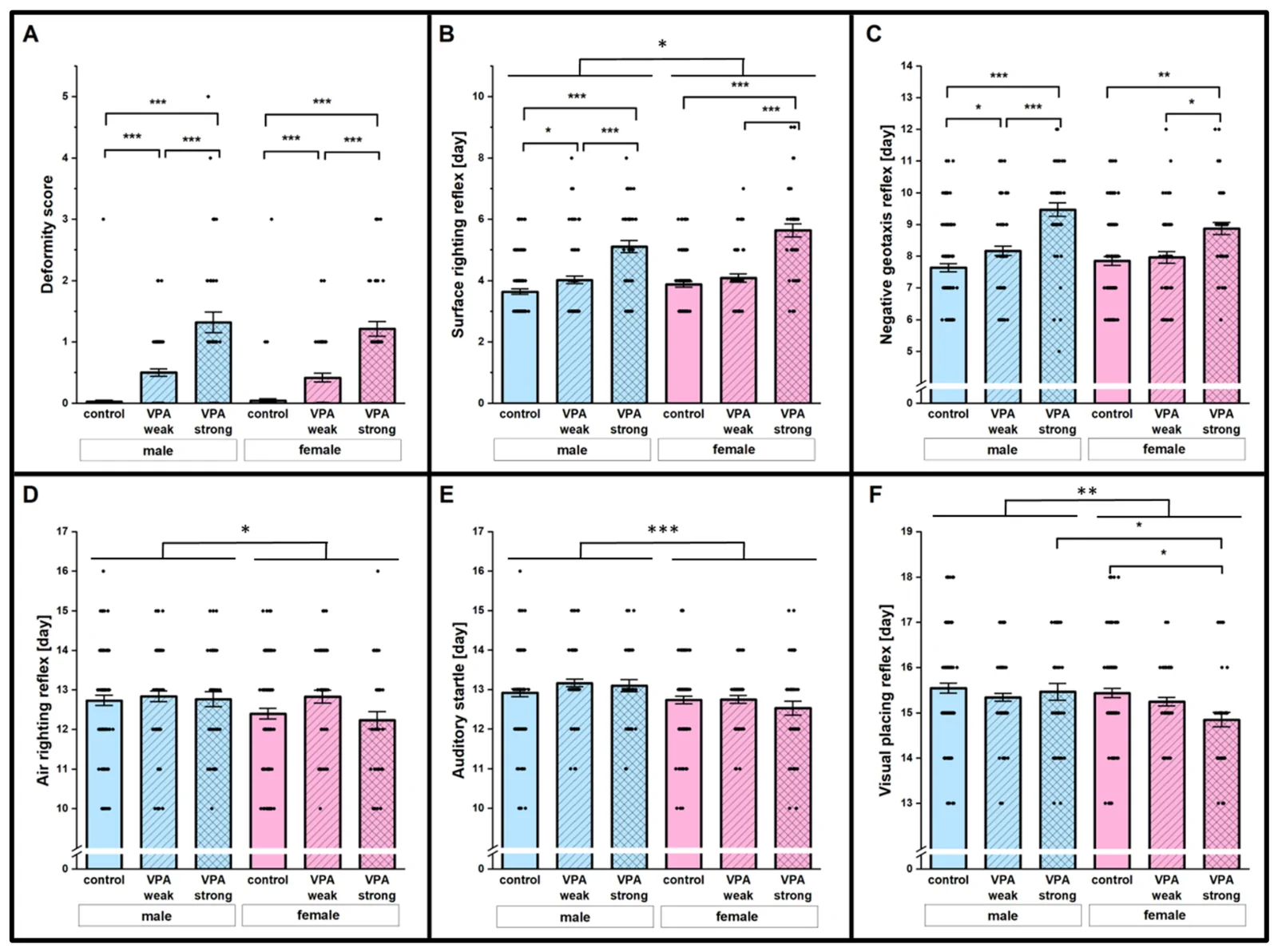
The effects of VPA on early sensorimotor development. (**A**) The deformity score was significantly increased by prenatal VPA treatment in both sexes, (**B**) the surface righting reflex and (**C**) negative geotaxis reflex appeared with a delay in the VPA-treated pups in both sexes, (**D**) the air righting reflex and (**E**) auditory startle reflex were not significantly affected by VPA treatment, and (**F**) in the visual placing reflex, differences emerged between the sexes. The data are expressed as the mean value ± SEM (statistical analysis: two-way ANOVA; * means significant difference between two groups; *: *p* < 0.05, **: *p* < 0.01, ***: *p* < 0.001).

Morphological alterations (deformities such as tail kink or toe abnormalities) were very rare in the control rat pups (i.e., born from the dams treated with saline during pregnancy). VPA treatment during pregnancy caused a significant increase in the frequency of such deformities in the offspring of both sexes (Figure 4A). The subgroup separated as “strong VPA effect”, based on the PCA, had a significantly higher deformity score than the other groups in both sexes.

The surface righting reflex was carried out by the rat pups on P3–6, which was significantly delayed by VPA in both sexes, although in the case of the female offspring, only the “VPA strong” subgroup differed significantly from controls. The appearance of the surface righting reflex showed sex-dependence; the male pups could perform it significantly earlier compared to the females (Figure 4B). For the appearance of the negative geotaxis reflex (first carried out successfully on P6–11), the effect of VPA was exactly the same as for the surface righting reflex, and this reflex appeared at the same age in both sexes (Figure 4C). The air righting reflex appeared somewhat later in development, around P10–16. Here, prenatal VPA treatment did not cause any significant delay in its manifestation, although among the female pups, the “VPA weak” group showed a tendency to delay, while the “VPA strong” group showed an early appearance of the reflex. Overall, the female pups performed this reflex significantly earlier compared to the males (Figure 4D). The auditory startle reflex, which appeared at the same period of development, was not significantly affected by prenatal VPA treatment but showed sex dependence: it appeared earlier in the female pups compared to the males (Figure 4E). The visual placing reflex requires the openness of the eyes and appeared around P13–18. In the males, prenatal VPA treatment did not influence the appearance of this reflex. In the female pups, the “VPA strong” group could carry out this reflex significantly earlier than the control females or the male “VPA strong” group. In addition, sex-dependence could be detected: the females could perform the reflex earlier than the males (Figure 4F).

#### 3.2.2. Repetitive Behavior

**Figure 5:**
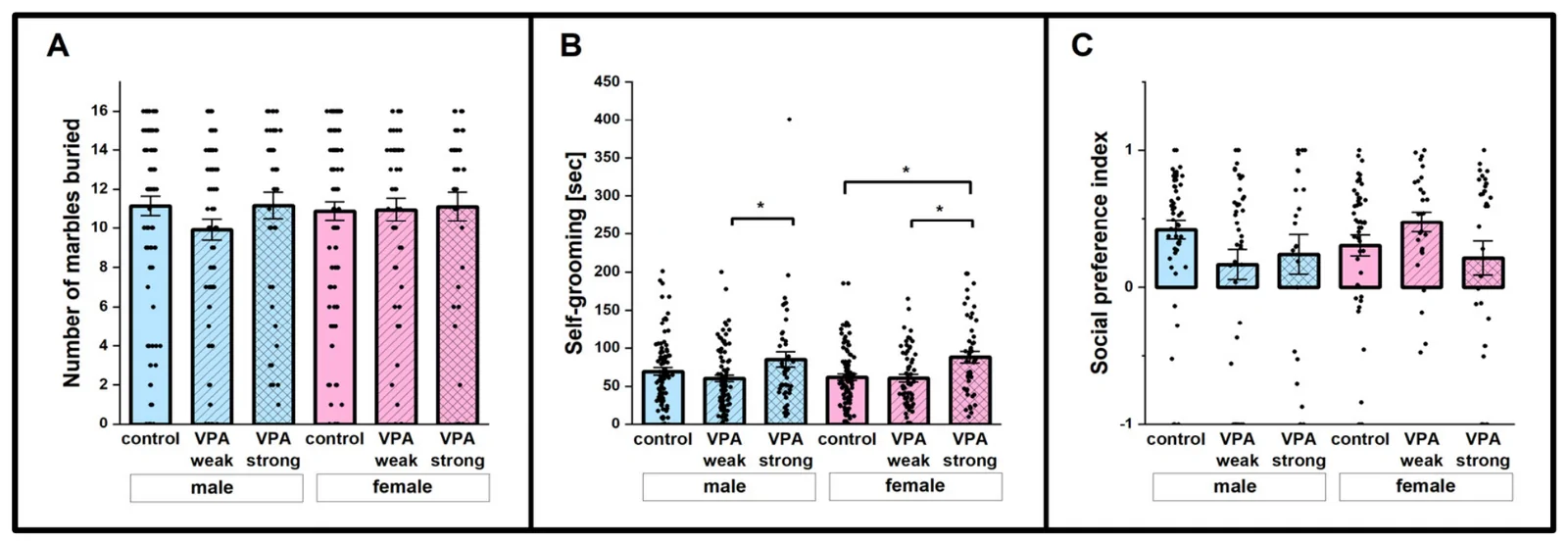
The effects of VPA on repetitive and social behavior. (**A**) Marble burying behavior was not affected by prenatal VPA treatment in either sex. (**B**) Self-grooming behavior increased in a subgroup of VPA-treated rats in both sexes. (**C**) The social preference index showed a tendency to decrease in all of the VPA-treated males, while the females seemed less affected: one VPA-treated subgroup displayed slightly higher, while the other displayed slightly lower, sociability than the controls. The data are expressed as the mean value ± SEM (statistical analysis: two-way ANOVA; * means significant difference between two groups; *: *p* < 0.05).

The number of marbles buried during the test was highly variable in all of the treatment groups. Prenatal VPA treatment did not cause a significant alteration in this parameter (Figure 5A). Time spent with self-grooming is another measure of compulsive, repetitive behavior. Prenatal VPA treatment led to significantly increased self-grooming in subgroups of animals of both sexes (“VPA strong” groups), while the behavior in the “VPA weak” subgroup did not differ from the controls (Figure 5B).

#### 3.2.3. Social Behavior

The social preference index is calculated based on the time spent exploring a cage containing a fellow rat vs. an empty cage in a three-chamber apparatus. The control rats of both sexes displayed a clear preference towards the social stimulus. Prenatal VPA treatment caused a visible tendency toward a decrease in social preference in both of the male groups, although the social preference index was not significantly lower compared to the controls. In the females, one VPA-treated subgroup (“VPA weak”) displayed a tendency toward higher social preference than the controls, while the other subgroup (“VPA strong”) seemed to have lower sociability, similarly to their male counterparts (Figure 5C).

### 3.3. The Excitability of the Medial Prefrontal Cortex Is Influenced in a Sex-Dependent Manner

#### 3.3.1. Afterdischarges Evoked by High-Frequency Stimulation

**Figure 6:**
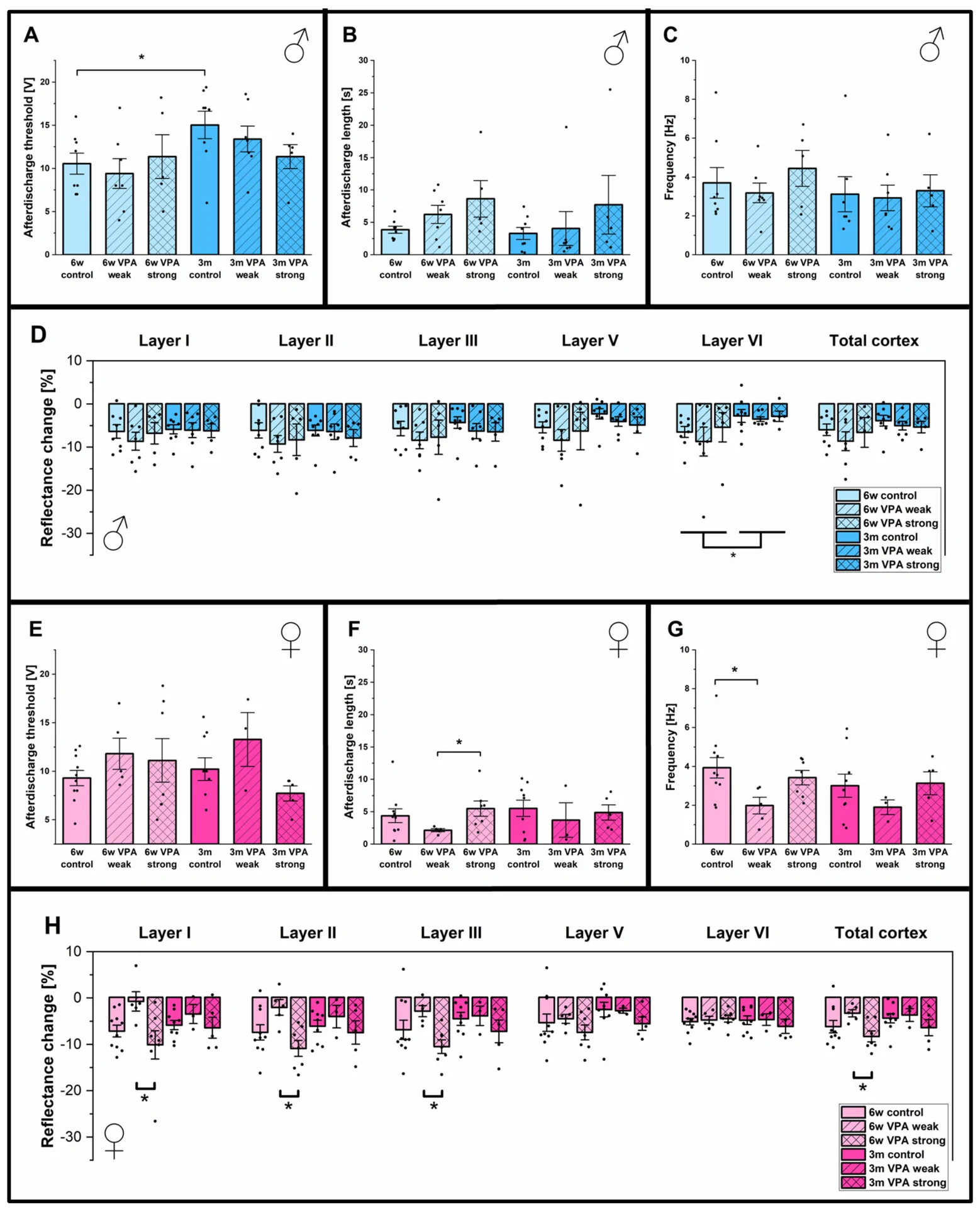
Medial prefrontal cortex excitability measured with afterdischarges. (**A**) The afterdischarge threshold, (**B**) afterdischarge length, (**C**) frequency, and (**D**) IOS reflectance change in the male groups. (**E**) The afterdischarge threshold, (**F**) afterdischarge length, (**G**) frequency, and (**H**) IOS reflectance change in the female groups. The data are expressed as the mean value ± SEM. N = 8, 7, 5, 8, 7, and 5 in the case of the males, and N = 10, 5, 7, 9, 3, and 5 in the case of the females in the 6-week-old control, VPA weak, and VPA strong and the 3-month-old control, VPA weak, and VPA strong groups, respectively (statistical analysis: three-way ANOVA; * means significant difference between two groups; *: *p* < 0.05).

The excitability of cortical microcircuits was examined in acute brain slices prepared from 6-week-old and 3-month-old rats of both sexes, representing adolescent and adult age. Network excitability may be characterized by the readiness of the slice to generate ADs as a response to repetitive high-frequency stimulation of relatively high intensity [30]. In the male rats, the voltage threshold for evoking ADs was lower in the 6-week-old controls compared to the 3-month-old ones, reflecting a higher excitability of the adolescent mPFC (Figure 6A). In the females, no such clear age dependence could be detected (Figure 6E). VPA treatment did not cause significant alterations in the AD threshold. AD length and burst frequency also depend on the excitability and the connectivity of cortical microcircuits; however, these parameters did not show significant age dependence (Figure 6B,C,F,G). In the male rats, VPA treatment tended to increase AD length in both age groups; the strength of the effect correlated well with the subgroups based on the behavioral data (Figure 6B). On the other hand, in the females, the data from the “VPA weak” group suggested decreased mPFC excitability compared to the controls, while the “VPA strong” group showed tendencies toward increased excitability (Figure 6F,G). The intrinsic optical signal (change in reflectance) usually correlates with the electrical activity of brain slices [31].

The analysis of an ROI spanning the whole cortex allows examination of the role of different cortical layers in excitability. In layer V, an age-related effect was observed: reflectance decreased more intensely in the 6-week-old groups than in the older groups (*p* = 0.03). In layer I, the interaction between sex and treatment was significant (*p* = 0.04), meaning that the treatment effect was different in males and females, while in layer VI, the interaction between age and sex was significant (*p* = 0.03). In the males, the slices coming from the 3-month-old rats displayed significantly lower IOS activity in layer VI of the cortex. There are no significant differences in IOS values in the other layers or among the treatment groups (Figure 6D), but the tendencies in the change in reflectance mirror the data obtained with electrophysiology (higher excitability in the younger age and in the VPA-treated groups). The contribution of infragranular layers seems to decrease with age. In the 6-week-old females (Figure 6H), the mPFC slices coming from the “VPA weak” rats displayed significantly lower reflectance change compared to the “VPA strong” group, mostly in the supragranular layers.

#### 3.3.2. Spontaneous Activity in MFR

**Figure 7:**
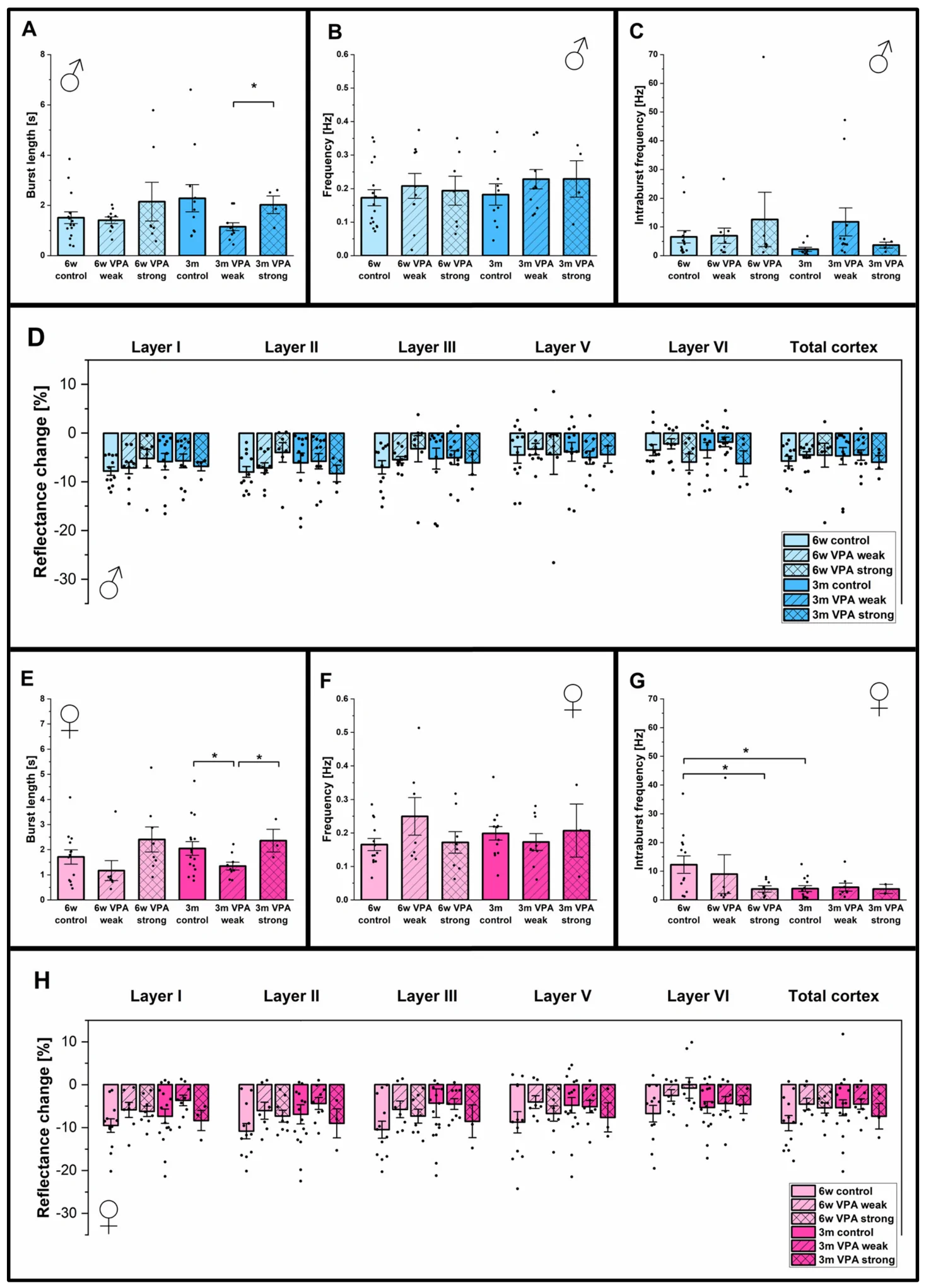
Medial prefrontal cortex seizure susceptibility measured with MFR. (**A**) Burst length, (**B**) frequency, (**C**) intraburst frequency, and (**D**) IOS reflectance change in the male groups. (**E**) Burst length, (**F**) frequency, (**G**) intraburst frequency, and (**H**) IOS reflectance change in the female groups. The data are expressed as the mean value ± SEM. (**A**,**D**) N = 16, 10, 7, 11, 11, and 4 in the case of the males, and N = 13, 7, 8, 16, 9, and 3 in the case of the females; (**B**) N = 16, 10, 7, 10, 11, and 4 in the case of the males, and N = 13, 7, 8, 13, 9, and 3 in the case of the females; and (**C**) N = 14, 9, 7, 9, 11, and 4 in the case of the males. and N = 13, 6, 8, 13, 8, and 2 in the case of the females in the 6-week-old control, VPA weak, and VPA strong, and the 3-month-old control, VPA weak, and VPA strong groups, respectively (statistical analysis: three-way ANOVA; * means significant difference between two groups; *: *p* < 0.05).

Magnesium-free solution is used to evoke epileptiform discharges in brain slices as an in vitro model of seizure susceptibility. Spontaneous network activity develops in cortical structures usually 20–30 min after starting perfusion with MFR, due to the removal of the Mg^2+^ blockade from NMDA-type glutamate receptors [32]. The latency time to first ictal-like activity was similar in both sexes and all of the age and treatment groups (Figure S1). VPA treatment had a significant effect independent of sex and age: the burst lengths in the “VPA weak” groups were significantly smaller compared to the control groups (*p* = 0.02), while the bursts of the “VPA strong” groups were significantly longer compared to the “VPA weak” groups (*p* = 0.005). In the male rats, there were no significant differences among age or the treatment groups in the parameters for epileptiform bursts. However, in the 3-month-old “VPA weak” subgroup, there was a tendency toward decreased burst length compared to the controls, with a parallel increase in burst frequency and intraburst frequency (Figure 7A–C). Thus, the pattern of the spontaneous activity changed, but there were no differences in the intrinsic optical signal, suggesting that the overall intensity of the activity was the same in these groups (Figure 7D).

In the females, there was an age-dependent decrease in the intraburst frequency of epileptiform bursts (Figure 7G). Among the 6-week-old animals, the “VPA strong” subgroup displayed a similarly lower intraburst frequency compared to the age-matched controls. In the older age group, the “VPA weak” treated slices showed a lower burst length compared to controls (Figure 7E), without a difference in burst frequency (Figure 7F). No consistent age- or treatment-dependent change was seen in the IOS signal in the mPFC slices from the female rats (Figure 7H).

### 3.4. Subtle Alterations in the Excitability of Lateral Entorhinal Cortex Occur in the VPA-Treated Rats

#### 3.4.1. Afterdischarges Evoked by High-Frequency Stimulation

In the lateral entorhinal cortex (LEC), there was a significant interaction between treatment, sex, and age for intraburst frequency values (*p* = 0.045), meaning that the three factors affect this parameter not independently of each other. Intraburst frequency values of the “VPA strong” groups were significantly lower compared to the control groups (*p* = 0.02).

In the case of IOS detection, a sex difference was observed in layers V and VI: the females had a greater decrease in reflectance than the males (layer V: *p* = 0.045, layer VI: *p* = 0.033). The effect of VPA treatment was also significant in these layers: the IOS decreased more intensely in the “VPA strong” groups than in the control groups (layer V: *p* = 0.018, layer VI: *p* = 0.049), while in layer VI, the value of the “VPA weak” groups was also more negative compared to the controls (*p* = 0.035).

**Figure 8:**
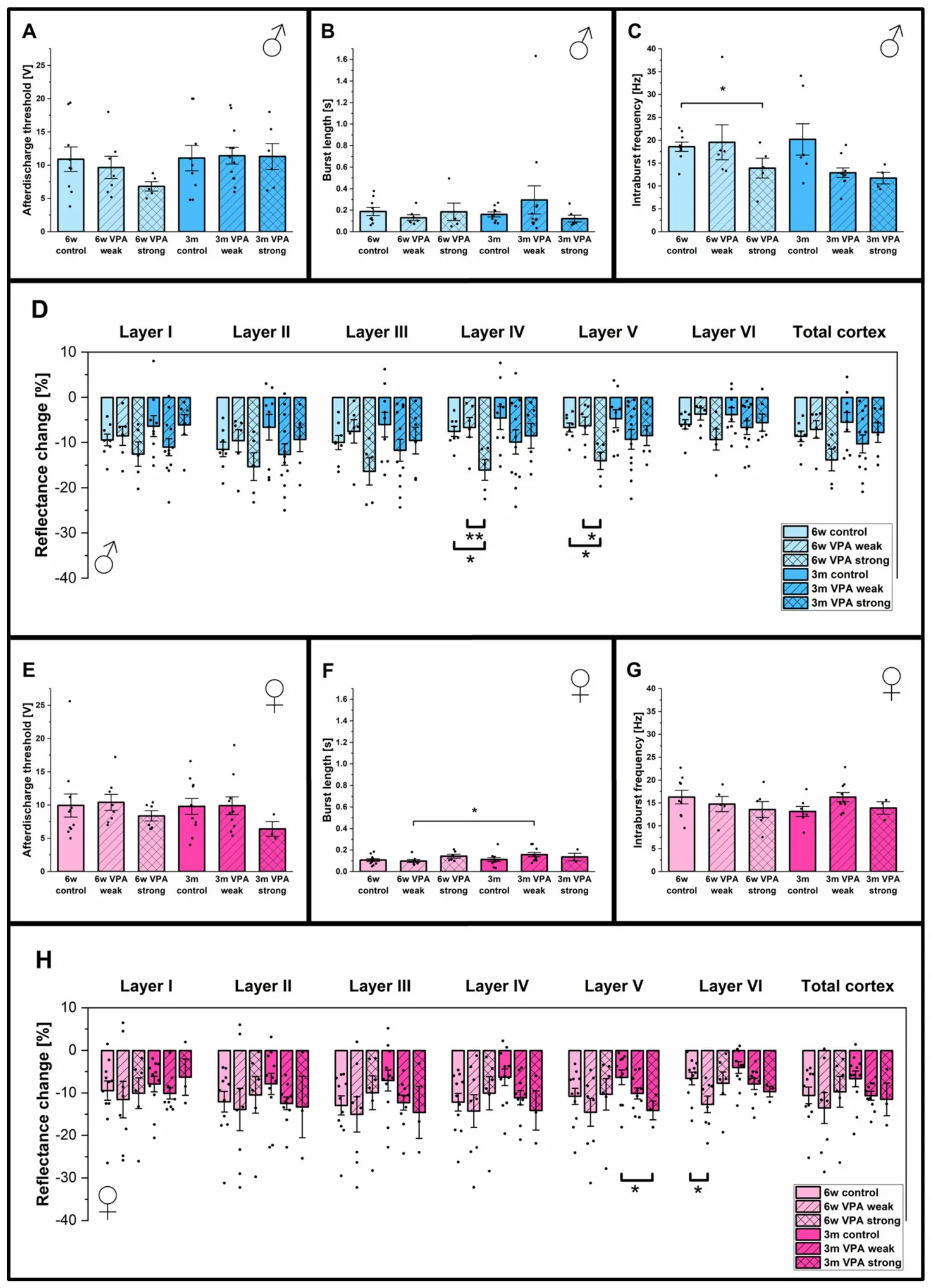
Lateral entorhinal cortex excitability measured with afterdischarges. (**A**) The afterdischarge threshold, (**B**) burst length, (**C**) intraburst frequency, and (**D**) IOS reflectance change in the male groups. (**E**) The afterdischarge threshold, (**F**) burst length, (**G**) intraburst frequency, and (**H**) IOS reflectance change in the female groups. The data are expressed as the mean value ± SEM. (**A**,**B**,**D**) N = 9, 7, 5, 9, 12, and 6 in the case of the males, and N = 11, 8, 6, 11, 10, and 3 in the case of the females; and (**C**) N = 9, 6, 5, 7, 10, and 4 in the case of the males, and N = 9, 5, 6, 7, 10, and 3 in the case of the females in the 6-week-old control, VPA weak, and VPA strong and the 3-month-old control, VPA weak, and VPA strong groups, respectively (statistical analysis: three-way ANOVA; * means significant difference between two groups; *: *p* < 0.05, **: *p* < 0.01).

In contrast to the mPFC, where slices originating from the adolescent males displayed higher excitability than the adult ones, in the case of the LEC, no age-dependent change in the AD threshold was detected in either sex (Figure 8A,E). Prenatal VPA treatment caused a tendency toward a threshold decrease and a significant decrease in the intraburst frequency of ADs in the “VPA strong” subgroup of the 6-week-old male rats compared to the controls, without a change in burst length (Figure 8B,C). Interestingly, this alteration was paralleled by a significant increase in the IOS signal in the infragranular layers of the LEC, suggesting more intense network activity during the whole recording (Figure 8D). The decrease in AD intraburst frequency persisted as a tendency in the 3-month-old male VPA groups, too, and a similar tendency toward an increase in the IOS could be observed.

In the females, similarly to the males, prenatal VPA treatment led to a tendency toward an AD threshold decrease in the LEC slices, mostly for the “VPA strong” group at the age of 3 months (Figure 8E). For this group, the IOS signal was significantly stronger in layer V compared to the controls (Figure 8H). AD burst length showed an age-dependent increase only in the “VPA weak” group (Figure 8F). In this group, higher IOS activity could be observed at 6 weeks of age in layer VI of the LEC compared to the control group (Figure 8H). However, there were no consistent changes in intraburst frequency (Figure 8G).

#### 3.4.2. Spontaneous Activity in MFR

Compared to the mPFC, in the LEC slices, longer epileptiform bursts developed, reflecting a difference in connectivity and excitability. The LEC was studied in horizontal brain slices, which preserve part of entorhinal–hippocampal connections, allowing for easier buildup of epileptiform activity. Latency time to first ictal-like activity was shorter than for the mPFC and showed sex-dependence: it was significantly shorter in the males compared to the females. However, it was similar for age and the treatment groups (Figure S1). Concerning epileptiform activity parameters in the LEC, in the case of burst length, the effect of treatment was significant: in the “VPA strong” groups, the length of the bursts was significantly shorter than in the control groups (*p* = 0.016). Similar to the AD measurements, there was a significant interaction among treatment, sex, and age for intraburst frequency values (*p* = 0.011), meaning that the three factors affect this parameter not independently of each other.

In the case of IOS recordings, a sex difference was observed in each layer: the males had a greater decrease in reflectance than the females (layer I: *p* = 0.010, layer II: *p* = 0.015, layer III: *p* = 0.029, layer IV: *p* = 0.30, layer V: *p* = 0.007, and layer VI: *p* = 0.024, total cortex: *p* = 0.011). With the exception of layers V and VI, the treatment effect was also significant: the “VPA strong” groups had a slight decrease in reflectance compared to the control groups (layer I: *p* = 0.040, layer II: *p* = 0.010, layer III: *p* = 0.012, and layer IV: *p* = 0.36, total cortex: *p* = 0.021). With the exception of layers I and VI, less reflectance decrease was measured in the “VPA weak” groups compared to the control groups (layer II: *p* = 0.048, layer III: *p* = 0.047, layer IV: *p* = 0.33, and layer V: *p* = 0.018, total cortex: *p* = 0.039).

**Figure 9:**
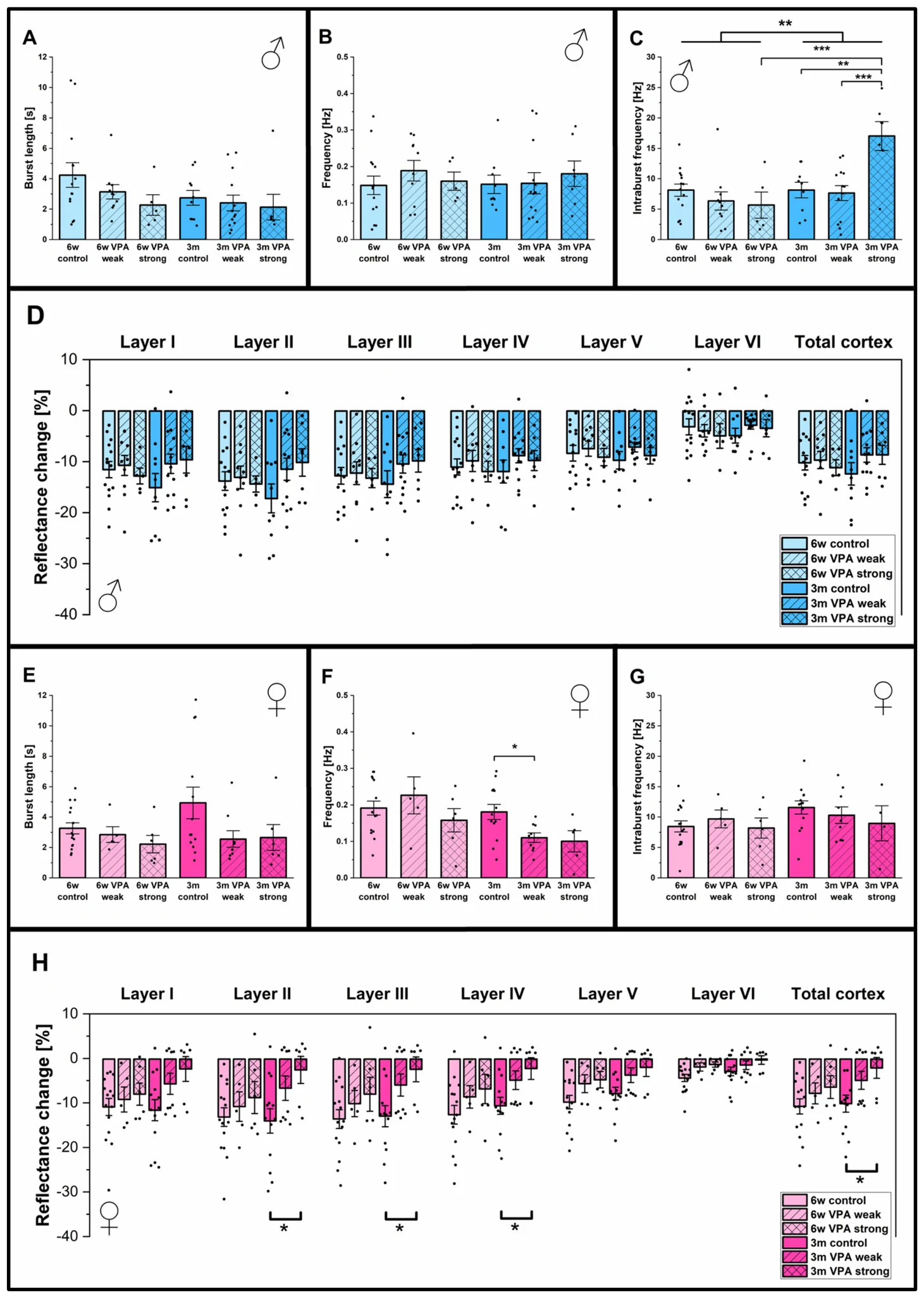
Lateral entorhinal cortex seizure susceptibility measured with MFR. (**A**) Burst length, (**B**) frequency, (**C**) intraburst frequency, and (**D**) IOS reflectance change in the male groups. (**E**) Burst length, (**F**) frequency, (**G**) intraburst frequency, and (**H**) IOS reflectance change in the female groups. The data are expressed as the mean value ± SEM. (**A**,**D**) N = 14, 10, 5, 10, 13, and 7 in the case of the males, and N = 15, 5, 6, 13, 9, and 6 in the case of the females; (**B**) N = 14, 10, 5, 9, 13, and 7 in case of males, and N = 15, 5, 6, 13, 9, and 5 in the case of the females; and (**C**) N = 14, 10, 5, 9, 13, and 7 in the case of the males, and N = 15, 5, 6, 13, 9, and 4 in the case of the females in the 6-week-old control, VPA weak, and VPA strong and the 3-month-old control, VPA weak, and VPA strong groups, respectively (statistical analysis: three-way ANOVA; * means significant difference between two groups; *: *p* < 0.05, **: *p* < 0.01, ***: *p* < 0.001).

No age- or treatment-dependent changes could be observed in the male rats in burst length or frequency (Figure 9A,B). In contrast, the intraburst frequency was significantly higher in the 3-month-old rats compared to the 6-week-old ones. Intraburst frequency was also higher in the “VPA strong” group at 3 months of age compared to the age-matched controls (Figure 9C). There was no clear difference in IOS signal intensity according to the treatment groups, but a tendency toward decreased activity in the case of the 3-month-old VPA-treated groups seems to be present in most cortical layers (Figure 9D).

In LEC slices originating from the female rats, MFR-evoked epileptiform activity showed a tendency toward age-dependent change: the length of bursts and their internal frequency were higher in the 3-month-old group compared to the 6-week-old group, without any change in the frequency of bursts (Figure 9E–G). Prenatal VPA treatment caused a significant decrease in burst frequency in the 3-month-old “VPA weak” group, but similar decreasing tendencies could be seen in the “VPA strong group”, too (Figure 9F). Also, burst length tended to be lower in the 3-month-old VPA-treated rats than in the controls (Figure 9E). This decreased field potential activity correlated well with the IOS recorded in the LEC slices: in the 3-month-old “VPA strong” group, the reflectance change in the slices was significantly lower than that of the controls, mostly in the supragranular layers and layer IV (Figure 9H).

### 3.5. VPA Treatment Strengthens Correlation Between Behavior and Cortical Excitability

**Figure 10:**
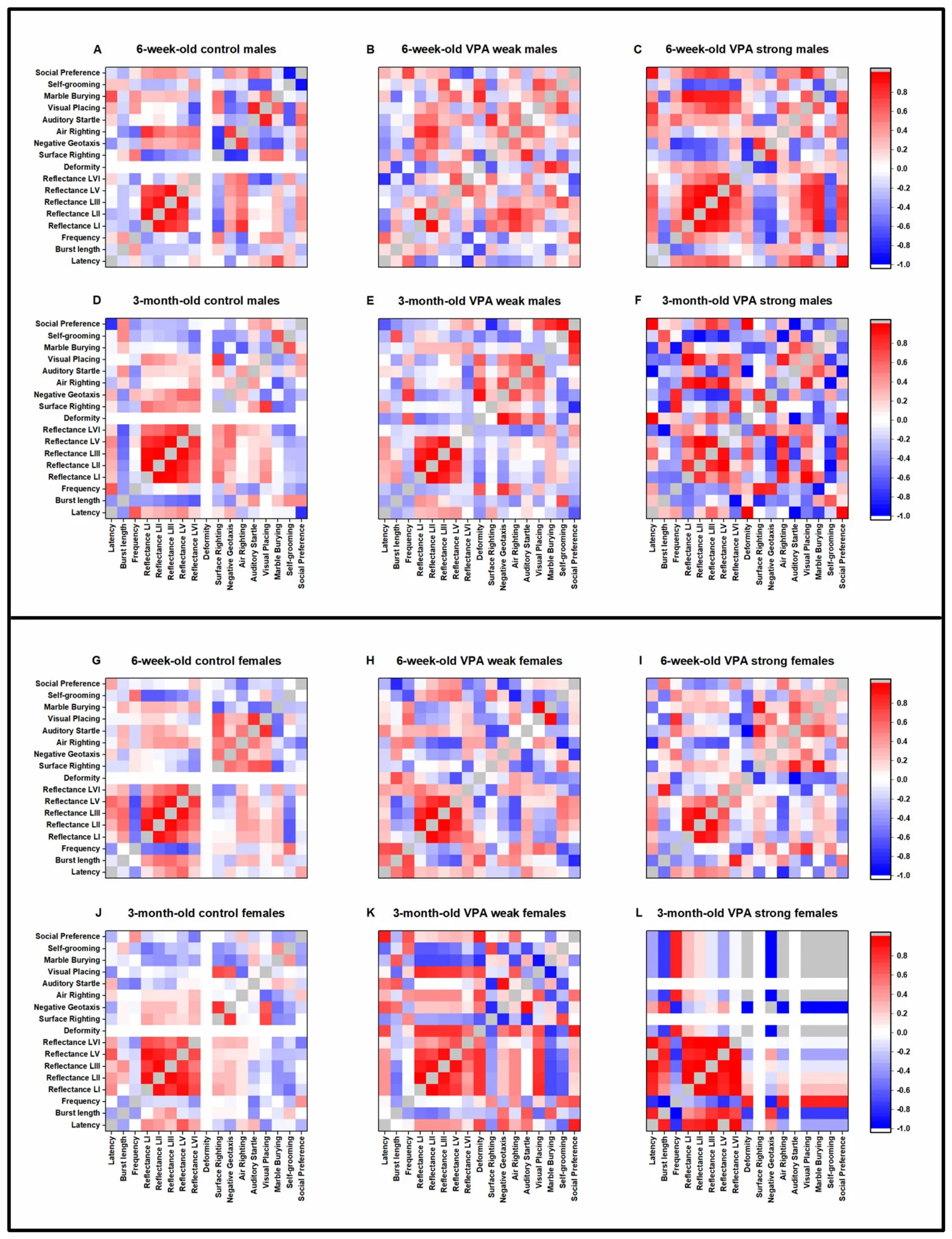
Correlation values between morphological, behavioral, and mPFC excitability parameters based on MFR-evoked spontaneous electrophysiological activity and IOS recording. Different panels show data for different treatments, sex, and age groups. Correlation values have been color-coded from red (+1) to blue (−1). Autocorrelation is marked with light gray squares, while lack of correlation due to missing data is marked with white. (**A**) 6-week-old control males (N = 16), (**B**) 6-week-old VPA weak males (N = 10), (**C**) 6-week-old VPA strong males (N = 7), (**D**) 3-month-old control males (N = 11), (**E**) 3-month-old VPA weak males (N = 11), (**F**) 3-month-old VPA strong males (N = 4), (**G**) 6-week-old control females (N = 13), (**H**) 6-week-old VPA weak females (N = 7), (**I**) 6-week-old VPA strong females (N = 8), (**J**) 3-month-old control females (N = 16), (**K**) 3-month-old VPA weak females (N = 9), and (**L**) 3-month-old VPA strong females (N = 3).

To examine the degree of association between all recorded parameters in the individual rats, pairwise Pearson’s correlation values were calculated for each treatment, sex, and age group. For this analysis, only the MFR-evoked spontaneous epileptiform activity paradigm was selected to assess cortical excitability data. Correlations including mPFC excitability parameters can be seen in Figure 10, while correlations with the LEC results are reported in Figure S2. The correlation values are visualized on a heatmap; the red color indicates positive, while the blue color indicates negative correlation. The significance of correlation values is reported in Tables S1 and S2.

In almost all correlation matrices in Figure 10, a conspicuous reddish square is visible, corresponding to IOS data belonging to different cortical layers, demonstrating positive correlation of reflectance change throughout the cortex. Correlation analysis also supports that field potential activity is associated with intrinsic optical activity in brain slices. A more intense decrease in reflectance value is paralleled by higher burst frequency or higher burst length, as shown by negative correlations between these parameters, e.g., in Figure 10A. Latency time to the first epileptiform burst shows an inverse association with IOS strength, shown by positive correlations with reflectance values (e.g., Figure 10C).

The main aim of our correlation analysis was to ascertain whether easily quantifiable deformities or behavioral data show any mathematical association with cortical excitability measures in later life in VPA-treated rats. Although the correlation coefficients are overall higher in the VPA-treated groups, as shown by the deeper colors of the heatmaps, the number of statistically significant pairwise correlations is not higher in the VPA-treated groups compared to the controls. This may be due to lower sample numbers in the treated groups. Thus, correlation analysis fails to consistently support our hypothesis that higher deformity score, more prominent sensorimotor developmental delay, higher repetitive behavior, or lower social preference will correspond to higher mPFC excitability, but some results are promising.

In the 6-week-old “VPA weak” male group, the results are controversial: deformity score positively correlates with marble burying behavior, but negatively correlates with the length of bursts, and the social preference index positively correlates with the frequency of bursts (Figure 10B). In the 6-week-old “VPA strong” male group, the latency time of bursts positively correlates with the social preference index, which aligns with our expectations, but other measures fail to support the relationship (Figure 10C).

The 3-month-old “VPA weak” male group yields more promising results: the deformity score shows significant positive correlations with the frequency of epileptiform bursts in the mPFC and also with the appearance time of negative geotaxis, air righting, and placing reflexes. The length of the bursts shows a significant positive correlation with grooming behavior (Figure 10E). The 3-month-old “VPA strong male group” shows controversial results again, which may be explained by the low sample number: the deformity score positively correlates with the social preference index and latency time of bursts, some unexpected findings. But latency time of bursts positively correlates with the social preference index, and grooming behavior negatively correlates with the reflectance value in layer II, which supports our expectations (Figure 10F).

In the case of the 6-week-old “VPA weak” female group, the appearance of the visual placing reflex displays a significant positive correlation with marble burying behavior and the frequency of bursts. On the other hand, the social preference index negatively correlates with the length of bursts (Figure 10H). These findings may indicate increased mPFC excitability in response to VPA, but overall, this effect could not be demonstrated compared to controls in females. In the 6-week-old “VPA strong” females, visual placing reflex appearance correlates positively with the frequency of bursts, just like in the other treatment groups. However, the deformity score shows a significant negative correlation with burst frequency and the appearance of auditory startle and surface righting reflexes; the latter is in contradiction to our expectations based on group data (Figure 10I).

In the 3-month-old “VPA weak” female subgroup, the deformity score displays significant positive correlations with the appearance of air righting and visual placing reflexes, and also with the latency time of bursts and reflectance change in layers I-V of the cortex. This may be well interpreted, as this group had a tendency toward lower mPFC activity compared to the control group. Marble burying also correlates negatively with reflectance changes, but also with the deformity score, air righting, and visual placing reflexes; the latter findings are difficult to explain, as the marble burying behavior of the VPA-treated groups did not differ from the controls (Figure 10K). The 3-month-old “VPA strong” female group contains only 3 individuals, and some data are missing, so no valid conclusions can be drawn here (Figure 10L).

## 4. Discussion

Prenatal VPA exposure has been shown to cause widespread alteration in the development of the nervous system, leading to ASD-like pathology in rodents. The imbalance of excitatory and inhibitory processes appears to be a key feature in ASD in general and in the VPA model, too. Nervous system function depends on both the excitability of individual neurons and their synaptic connectivity and circuit functions. Homeostatic plasticity is a prominent autoregulatory process, which allows neurons, and even extensive brain areas, to keep neuronal activity relatively stable. These autoregulatory feedback mechanisms undergo dynamic changes during development and fail in effectiveness, giving rise to ASD or other psychiatric disorders. The whole process is of high complexity, given that developmental timelines depend on the brain area and sex, and it is often difficult to unravel original pathological changes and the results of homeostatic compensatory processes triggered by the insult [8,33].

The aim of the current study was to analyze the effect of VPA on behavior and the excitability of two selected brain areas, the LEC and mPFC. Our goal was to take into account the variability in affectedness between the two sexes and among individual rats, and to correlate behavioral phenotype with underlying microcircuit functions. We performed a PCA-based analysis on the deformity and behavioral results and further divided the VPA-treated rats into weakly and strongly affected subgroups. We hypothesized that the excitability of brain slices originating from the rats displaying more severe morphological and behavioral alterations would differ more from controls than slices originating from less affected rats.

VPA induced morphological alterations (tail kink and sometimes toe abnormalities) in the rat pups of both sexes. Early sensorimotor development (until P10) was delayed in all of the VPA-treated male pups, but only in a subset of the female pups. Later sensorimotor functions (P10–16) were only affected in the females; in several cases, VPA treatment seemed to cause accelerated development. This is in contradiction with a previous study reporting a delay of later sensorimotor development in VPA-treated male rats. In that study, however, tail kink was observed in every treated pup, so they were affected to a greater degree by VPA treatment, as in our case [16]. According to another study reporting a very detailed examination of early postnatal physical and sensorimotor development in VPA-treated rat pups, the developmental trajectory is differentially affected in the two sexes, with males showing more severe motor deficits, females suffering more delay in physical development, and the development of sensory functions being affected in both sexes [34].

Social deficits were more prominent in the male rats, while only a subset of the VPA-treated females showed some social interaction deficit. Other studies in which VPA-treated offspring of both sexes were analyzed also report that primarily the males display social deficits [24,35], although using assays different from the three-chamber test, researchers could demonstrate social deficits in female rodents, too [25,36]. On the other hand, the occurrence of repetitive behavior (i.e., self-grooming) increased in a subset of the VPA-treated rats in both sexes equally. This finding is consistent with previous studies reporting that stereotypic behavior occurs in both sexes in the VPA model [25,37].

The mPFC is the brain area perhaps the most widely studied in the context of ASD, as it is considered a hub for the regulation of social behavior. In human patients with ASD, early-life overgrowth of this cortical area has been described, along with abnormal columnar structure, namely increased minicolumn width [5]. There is an increasing number of mPFC abnormalities have been reported in studies using the VPA rodent model of ASD. Anatomical alterations, like neuronal loss in layers II/III and layer V [24] and local dendritic hyperconnectivity [38] have been evidenced. In 2-week-old VPA-treated rats, lower intrinsic excitability and hyperconnectivity of layer V pyramidal neurons have been demonstrated, along with synaptic hyperplasticity [27]. Decreased excitability of layer III pyramidal neurons has also been demonstrated in VPA-exposed 6-week-old male rats with the dynamic patch clamp method, but these changes seem to level off by 3 months of age [39]. Enhanced long-term potentiation has been reported in VPA-exposed young adult male offspring, too, along with upregulated protein levels of certain glutamate receptor subunits [20].

The results of the current study support the idea that prenatal VPA treatment induces increased network excitability in the mPFC of male rats. There is a clear tendency toward increased length of electrically evoked afterdischarges in brain slices from both 6-week-old and 3-month-old male rats, with a more prominent difference from the controls in the “VPA strong” subgroup of the exposed rats. The longer duration of ADs in the treated groups is paralleled by a tendency toward higher reflectance change in IOS.

There is much less information available about the effect of prenatal VPA treatment on mPFC functions in females, although it seems that the development of individual neurons and networks is affected even if their behavior does not display as severe deficits as that of males. Evidence suggests that VPA interferes with the normal maturation of layer III pyramidal neurons: it accelerates the positive shift in resting membrane potential occurring between 6 weeks and 3 months of age and prevents the expression of HCN channels during this period [39]. In the current study, similar effects were observed on the network level: the intraburst frequency of epileptiform discharges evoked with MFR decreased with age (it is significantly higher in 6-week-old compared to 3-month-old female rat mPFC slices), and in the strongly affected VPA-exposed subgroup, similar values to those of older animals were observed, suggesting premature development.

However, the effect of prenatal VPA treatment on mPFC excitability in females is intriguingly complex. In more than one case, we observed that more prominent changes in excitability could be seen in the “VPA weak” subgroup than in the “VPA strong” group. In addition, opposite tendential effects were seen in the two treated subgroups: the AD length and burst frequency decreased significantly in the “VPA weak” subgroup, while they increased in the “VPA strong subgroup” in both age groups. We can speculate that VPA treatment causes increased network activity, leading to compensatory homeostatic processes and an overshoot in the “VPA weak” group, but these are insufficient in the “VPA strong” group.

The other examined cortical area, the LEC, has an important role in memory encoding, spatial orientation, sensory integration, and cognitive flexibility—functions often impaired in ASD. There is no direct evidence for altered structure or function of the LEC in human ASD, but it is very likely an affected area, partly due to connections with the hippocampus [40]. In the rodent VPA model, the LEC has been hardly studied. As reported by us, layer III pyramidal neuron intrinsic and spiking properties display similar alterations in VPA-exposed rats as neurons in the mPFC, but to a lesser degree [39]. In contrast, there are several papers about alterations in the hippocampus in the VPA rodent model, some of them yielding contradictory results. A study has reported enhanced performance of VPA-exposed male and female rats in the Morris water maze, the gold standard test for spatial navigation, along with increased cell density in most hippocampal subfields [36]. Other studies have reported impaired performance for VPA-exposed male rats in the Morris water maze [16,41]. Electrophysiological data about hippocampal neurons in the VPA model are also somewhat contradictory: according to a study, CA1 pyramidal neurons show higher excitability in treated male rats [41], but we did not observe any difference in the excitability of CA1 pyramidal neurons in the males, only in the females [42]. On the other hand, in the 6-week-old male VPA-exposed rats, significantly increased network excitability of the CA1 area could be demonstrated, which levelled off by 3 months of age [42]. In the current study, the LEC slices from the 6-week-old “VPA strong” subgroup of males showed a tendency toward a decreased AD threshold and increased optical signal in infragranular layers, supporting the idea of the hyperexcitability of the hippocampal formation. Intraburst frequency of ADs was significantly decreased in this group compared to the controls. This finding might be related to an increased degree of depolarization block in the underlying neuronal population. On the other hand, in the 3-month-old “VPA strong” subgroup, increased intraburst frequency and decreased IOS were seen for epileptiform activity evoked with MFR. In the female rats, the 3-month-old “VPA strong” subgroup displayed significantly decreased reflectance change compared to the control during epileptiform activity evoked with MFR, which is consistent with a tendency toward decreased burst length and burst frequency. To a lesser degree, in the younger age group and the “VPA weak” subgroup, the data also indicate decreased excitability, so overall we can state that in females, prenatal VPA treatment leads to decreased network excitability of the LEC. Thus, marked sex differences exist concerning the functional alteration of certain brain areas in different periods of postnatal life.

Perhaps the most intriguing findings of our study are the variable effects of VPA on cortical excitability in female rats, which differ markedly from the more unidirectional effects in males. Human ASD is characterized by marked sex differences in the incidence but also the manifestation of the disorder. The rat prenatal VPA model also exerts differential effects according to the sex of the pups. Besides genetic differences between the two sexes, there are several factors that may contribute to these phenomena. It has been demonstrated that the degree of transcriptional alterations induced by VPA is higher in males. Sexual hormones affect brain development in a complex manner during both the embryonic and postnatal periods. Among other factors, they influence glutamatergic and GABAergic transmission [21]. This may affect the excitation–inhibition balance, and also compensatory homeostatic processes may be different between the two sexes. Also, the study by Anshu and colleagues [34] reports higher individual variability in females compared to males, concerning the effect of VPA on early sensorimotor development (although cautiously because of the lower sample size in females).

There are still very few studies addressing the spectrum aspect of prenatal VPA treatment in rodents. In most cases, VPA-treated offspring are analyzed as a homogeneous group, and correlation between different parameters recorded in individual rats is not examined. An exception to this trend is a study on the correlation of behavioral alterations and functional connectivity data recorded with MRI in VPA rats. The authors report altered functional connectivity in cortico–cerebellar and salience networks as a result of VPA treatment, and they demonstrate that normal social behavior correlates negatively with cerebellar alterations [35]. Another study reveals that associations between early developmental milestones and locomotor activity at weaning are stronger in VPA-treated rats than in controls [34].

The correlation analysis carried out in the current study between morphological, behavioral, and mPFC excitability parameters yielded partially satisfactory results. As a precious “byproduct”, we may mention that significant and consistent correlations could be demonstrated between electrophysiological field potential parameters and IOS (reflectance) data, as well as between IOS data recorded in different cortical layers. As for the correlations between deformity, sensorimotor development, behavior, and cortical excitability, overall, the strength of correlations increases with the affectedness by VPA treatment (i.e., from “VPA weak” to “VPA strong” subgroup). Both in the 3-month-old male and female “VPA weak” groups, a significant correlation between a higher deformity score and delayed appearance of various reflexes could be seen. In case of males, these parameters were positively associated with cortical excitability parameters, indicating increased mPFC excitability in response to VPA affectedness, while in females, the opposite tendency could be observed. Correlation analysis in the younger age groups and the “VPA strong” groups was much less conclusive.

Overall, the current study reports that in male animals, medial prefrontal and entorhinal cortical excitability, measured with electrophysiological and optical methods, follows the subgrouping of VPA-treated rats well according to the PCA performed based on morphological and behavioral variables. Correlation analysis between parameters yields some promising, but somewhat inconclusive, results. In female offspring, the relationship of these variables appears to be even more complex. Therefore, more studies investigating brain alterations in VPA-treated female offspring are warranted. In conclusion, the rodent prenatal VPA model recapitulates the spectrum features of ASD well, but this makes it difficult to use for testing drug candidates or potential therapeutic targets. The selection of a subgroup of VPA-exposed rodents can be beneficial by decreasing the variability in such studies.

## Figures and Tables

**Table 1:** The eigenvalues of the correlation matrix.

	Eigenvalue	Percentage of Variance	Cumulative
1	1.94018	21.56%	21.56%
2	1.61936	17.99%	39.55%
3	1.09495	12.17%	51.72%
4	1.01896	11.32%	63.04%
5	0.85333	9.48%	72.52%
6	0.73932	8.21%	80.73%
7	0.66122	7.35%	88.08%
8	0.63519	7.06%	95.14%
9	0.43748	4.86%	100.00%

## Data Availability

The original data presented in this study are openly available in FigShare at https://figshare.com/s/d877841bf9987c451810 (accessed on 17 July 2026).

## Supplementary Materials

The following supporting information can be downloaded at: https://www.mdpi.com/article/10.3390/cells15171617/s1. Figure S1: Latency time for epileptiform activity with MFR perfusion; Figure S2: Correlation values among morphological, behavioral, and LEC excitability parameters based on MFR-evoked spontaneous electrophysiological activity and IOS recording; Table S1: Pearson correlation values among the morphological, behavioral, and mPFC excitability parameters based on MFR-evoked spontaneous electrophysiological activity and IOS recording; Table S2: Pearson correlation values among the morphological, behavioral, and LEC excitability parameters based on MFR-evoked spontaneous electrophysiological activity and IOS recording.

## Abbreviations

The following abbreviations are used in this manuscript:

ACSF	Artificial Cerebrospinal Fluid
AD	Afterdischarge
ASD	Autism Spectrum Disorder
HFS	High-frequency Stimulation
IOS	Intrinsic Optical Signal
LEC	Lateral Entorhinal Cortex
MFR	Magnesium-free Ringer
mPFC	Medial Prefrontal Cortex
P3	Postnatal day 3
PCA	Principal Component Analysis
ROI	Region of Interest
VPA	Valproate
