# Supplementary material for: Individual and Sex Differences in Behavior and Cortical Excitability in the Rat Valproate Model of Autism
Source: Cells. 2026 Sep 5;15(17):1617. doi: 10.3390/cells15171617 (PMC13564657; doi:10.3390/cells15171617)
Supplement: Supplementary file 1 [file cells-15-01617-s001.zip › cells-4472783-supplementary.pdf]

Supplementary material

Seizure susceptibility was examined with MFR perfusion in prefrontal and entorhinal cortical slices. Latency time measured from the start of the perfusion with the convulsant until the first ictal-like activity was quantified, in addition to parameters describing the pattern of epileptiform activity (burst length, burst frequency, and intraburst frequency). Latency time values were consistent with the literature data reporting that 20-30 min was required for epileptiform activity to develop in this model in cortical slices. In the mPFC slices, latency times were markedly higher compared to the LEC slices. However, within one brain area, latency times were similar in both sexes and all age and VPA treatment groups, and only in the LEC were latency times significantly higher in the female groups compared to the males (p=0.022). (Fig. S1).

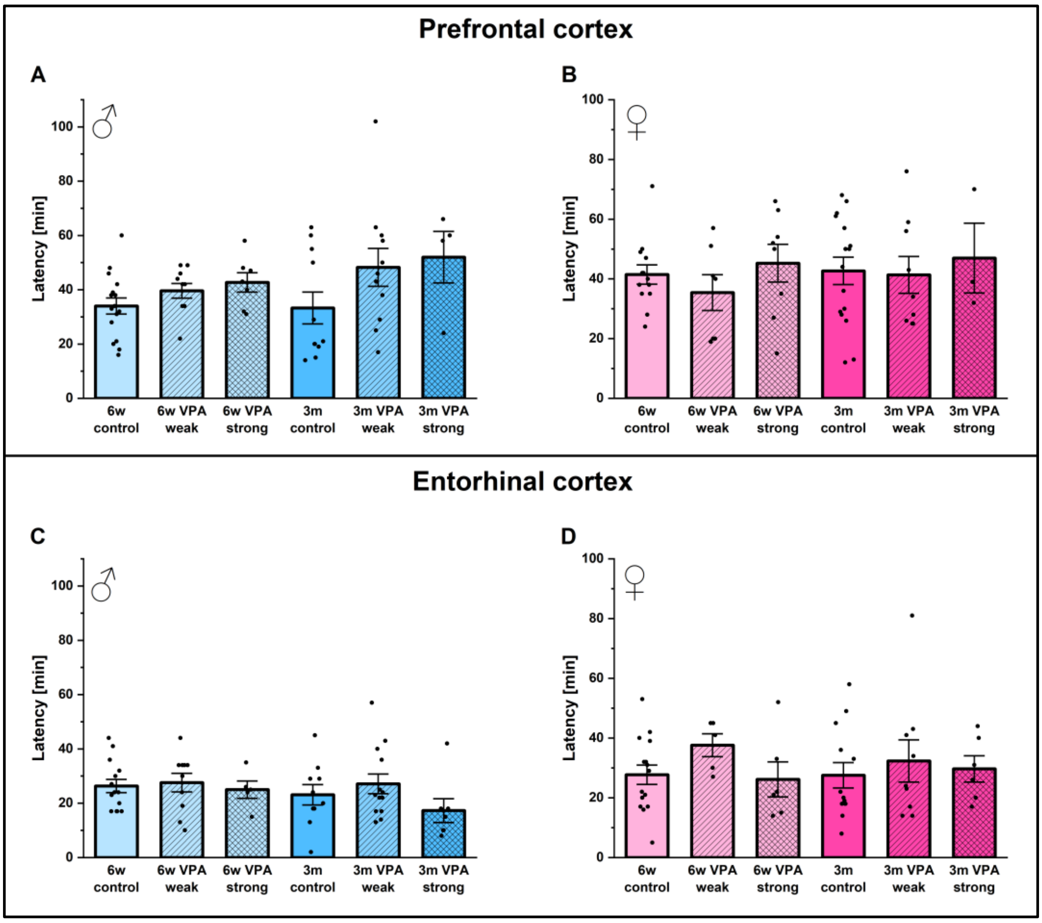

Figure S1. Latency time for epileptiform activity with MFR perfusion. **(A)** The medial prefrontal cortex, males, **(B)** the medial prefrontal cortex, females, **(C)** the lateral entorhinal cortex, males, and **(D)** the lateral entorhinal cortex, females. The data are expressed as the mean value ± SEM. (A): N = 16, 10, 7, 11, 11, and 4; (B) N = 13, 7, 8, 16, 9, and 3; (C): N = 14, 10, 5, 10, 13, and 7; and (D) N = 15, 5, 6, 13, 9, and 6 for the 6-week-old control, VPA weak, and VPA strong, and the 3-month-old control, VPA weak, and VPA strong groups, respectively. (Statistical analysis: three-way ANOVA).

Pairwise correlation analysis was performed among all of the recorded parameters in the different treatment, age and sex groups. The correlation analysis including mPFC excitability data is reported in the main manuscript (Fig. 10). The significance values for these correlations are summarized below, in Table S1.

Correlation analysis was also performed using the LEC excitability data, and the results are shown in Fig. S2. Unfortunately, for the 6-week-old control male rats, whose LEC brain slices were

tested with MFR for excitability, all behavioral data are missing (Fig. S2A). The significance values for the above correlations are summarized below, in Table S2.

Similar to Fig. 10, a reddish square is visible in all correlation matrixes corresponding to the IOS data from the different cortical layers, demonstrating positive correlation of reflectance change throughout the LEC. In the male groups, the latency time to the first epileptiform burst positively correlates with relative reflectance change, indicating that shorter latency times lead to more intense cortical activity in male rats. In contrast, a negative correlation is observed in several of the treated female groups, indicating that the appearance time of bursts does not necessarily correlate with the intensity of cortical epileptiform activity measured with IOS.

In the 6-week-old "VPA weak" male group, the number of buried marbles positively correlates with the deformity score, while negative geotaxis negatively correlates with deformity and the marble burying test result (Fig. S2B). The length of the bursts shows significant positive correlation with air righting and visual placing reflex in the 6-week-old "VPA strong" male group. Several behavioral parameters are positively correlated with each other: the surface and air righting reflex with negative geotaxis, and air righting with the visual placing reflex. However, the time spent on self-grooming negatively correlates with burst frequency and the auditory startle reflex, and an increase in the number of buried marbles is associated with an increase in the social preference index (Fig. S2C).

In the group of the 3-month-old "VPA weak" males, burst length negatively correlates with changes in IOS, consistent with increased excitability in this group compared to controls. Negative geotaxis and social preference index positively correlate with visual placing reflex. However, controversial results can be observed in this group: the social preference index negatively correlates with burst latency, as well as time spent on self-grooming with the appearance of auditory startle reflex (Fig. S2D). In the 3-month-old "VPA strong" male group, the results are controversial: the deformity score and the results for the air righting reflex negatively correlate with reflectance change in the supragranular layers, but the burst length negatively correlates with negative geotaxis, auditory startle and visual placing reflex tests. The social preference index negatively correlates with the appearance of auditory startle and the number of buried marbles, consistent with reduced sociability in this treated group (Fig. S2F).

In the 6-week-old "VPA weak" female group, a more intense decrease in reflectance value in layer III and layer IV is paralleled with higher burst length, and the latency time to the first epileptiform burst positively correlates with the social preference index and burst frequency with the appearance of the air righting reflex. In contrast, burst frequency negatively correlates with the deformity score and the number of buried marbles, the burst length positively correlates with the appearance of the air righting reflex, and negative geotaxis positively correlates with the reflectance change in layers I-IV (Fig. S2H). In the 6-week-old "VPA strong" female group, the decrease in the reflectance value in layer VI is paralleled by a higher burst frequency (negative correlation), but positively correlates with the appearance of the surface righting reflex (Fig. S2I).

In the 3-month-old "VPA weak" female group, the deformity score positively correlates with surface righting reflex test and negatively correlates with the social preference index. Time spent on self-grooming positively correlates with the air righting reflex, while marble burying negatively correlates with auditory startle in this group (Fig. S2K). In the 3-month-old "VPA strong" female group, the length of bursts negatively correlates with the social preference index, suggesting an

association between higher excitability and reduced sociability (Fig S2L).

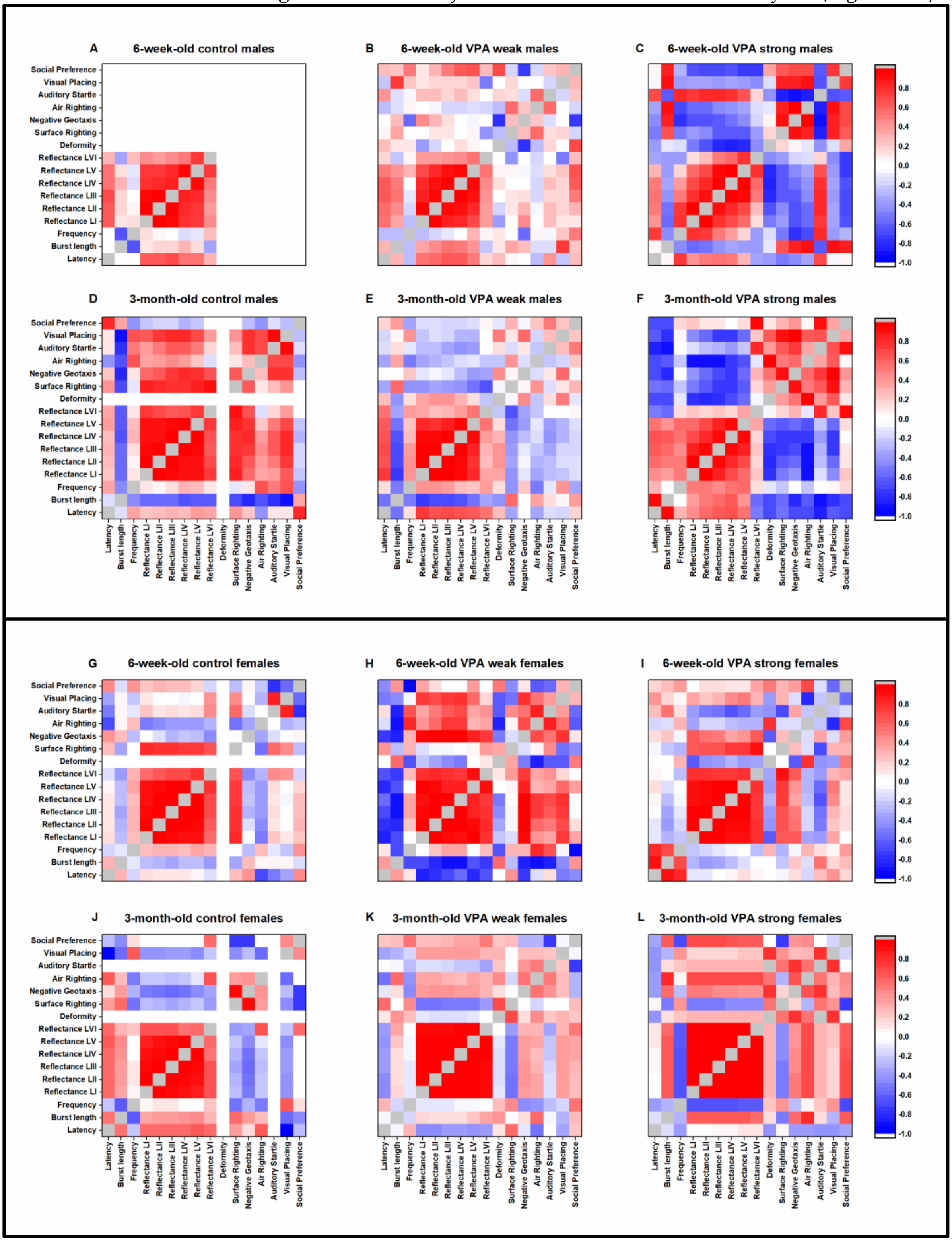

Figure S2. Correlation values among morphological, behavioral, and LEC excitability parameters based on MFR-evoked spontaneous electrophysiological activity and IOS recording. Different panels show data for different treatment, sex, and age groups. Correlation values have been color-coded from red (+1) to blue (-1). Autocorrelation is marked with light gray squares, while lack of correlation due to missing data are marked with white. **(A)** 6-week-old control males (N=14), **(B)** 6-week-old VPA weak males (N=10), **(C)** 6-week-old VPA strong males (N=5), **(D)** 3-month-old control males (N=10), **(E)** 3-month-old VPA weak males (N=13), **(F)** 3-month-old VPA strong males (N=7), **(G)** 6-week-old control females (N=15), **(H)** 6-week-old VPA weak females (N=5), **(I)** 6-week-old VPA strong females (N=6), **(J)** 3-month-old control females (N=13), **(K)** 3-month-old VPA weak females (N=9), and **(L)** 3-month-old VPA strong females (N=6).

Table S1. Pearson correlation values among the morphological, behavioral, and mPFC excitability parameters based on MFR-evoked spontaneous electrophysiological activity and IOS recording. The significant values are marked with red, autocorrelations with light gray. The correlation is significant at the 0.05 level. (Lat=latency, Bh=burst length, Freq=frequency, R=reflectance, Def=deformity score, SRR=surface righting reflex, NGR=negative geotaxis reflex, ARR=air righting reflex, AS=auditory startle, VPR=visual placing reflex, MB=marble burying, SG=self-grooming, and SP=social preference)

**6-week-old control male (N=16)**	**Lat**	**Bh**	**Freq**	**R LI**	**R LII**	**R LIII**	**R LV**	**R LVI**	**Def**	**SRR**	**NGR**	**ARR**	**AS**	**VPR**	**MB**	**SG**	**SP**
Latency	1.00	-0.16	0.15	-0.19	-0.29	-0.07	0.03	0.20	--	0.03	-0.42	-0.04	0.20	0.30	0.66	0.22	-0.13
Burst Length	-0.16	1.00	0.40	-0.24	-0.21	-0.21	-0.21	-0.09	--	0.19	-0.15	-0.43	-0.25	-0.17	-0.16	0.23	-0.32
Frequency	0.15	0.40	1.00	-0.43	-0.17	-0.02	-0.14	-0.45	--	0.51	-0.25	-0.58	0.04	0.39	0.41	-0.17	0.10
Reflectance LI	-0.19	-0.24	-0.43	1.00	0.88	0.78	0.70	0.00	--	-0.61	0.37	0.78	0.03	-0.03	0.22	-0.30	0.33
Reflectance LII	-0.29	-0.21	-0.17	0.88	1.00	0.91	0.73	-0.25	--	-0.53	0.32	0.57	0.11	0.05	0.22	-0.31	0.43
Reflectance LIII	-0.07	-0.21	-0.02	0.78	0.91	1.00	0.90	-0.10	--	-0.39	0.32	0.45	-0.01	0.01	0.26	-0.22	0.43
Reflectance LV	0.03	-0.21	-0.14	0.70	0.73	0.90	1.00	0.23	--	-0.33	0.40	0.51	-0.22	-0.17	0.23	-0.09	0.28
Reflectance LVI	0.20	-0.09	-0.45	0.00	-0.25	-0.10	0.23	1.00	--	-0.27	0.46	0.52	-0.52	-0.71	-0.31	0.38	-0.24
Deformity	--	--	--	--	--	--	--	--	--	--	--	--	--	--	--	--	--
Surface Righting	0.03	0.19	0.51	-0.61	-0.53	-0.39	-0.33	-0.27	--	1.00	-0.77	-0.75	0.11	0.51	0.53	-0.08	-0.33
Negative Geotaxis	-0.42	-0.15	-0.25	0.37	0.32	0.32	0.40	0.46	--	-0.77	1.00	0.75	-0.34	-0.59	-0.63	-0.12	0.42
Air Righting	-0.04	-0.43	-0.58	0.78	0.57	0.45	0.51	0.52	--	-0.75	0.75	1.00	-0.28	-0.47	-0.25	-0.10	0.28
Auditory Startle	0.20	-0.25	0.04	0.03	0.11	-0.01	-0.22	-0.52	--	0.11	-0.34	-0.28	1.00	0.84	0.13	-0.62	0.57
Visual Placing	0.30	-0.17	0.39	-0.03	0.05	0.01	-0.17	-0.71	--	0.51	-0.59	-0.47	0.84	1.00	0.57	-0.60	0.41
Marble Burying	0.66	-0.16	0.41	0.22	0.22	0.26	0.23	-0.31	--	0.53	-0.63	-0.25	0.13	0.57	1.00	-0.04	-0.16
Self-grooming	0.22	0.23	-0.17	-0.30	-0.31	-0.22	-0.09	0.38	--	-0.08	-0.12	-0.10	-0.62	-0.60	-0.04	1.00	-0.88
Social Preference	-0.13	-0.32	0.10	0.33	0.43	0.43	0.28	-0.24	--	-0.33	0.42	0.28	0.57	0.41	-0.16	-0.88	1.00

**6-week-old VPA weak male (N=10)**	**Lat**	**Bh**	**Freq**	**R LI**	**R LII**	**R LIII**	**R LV**	**R LVI**	**Def**	**SRR**	**NGR**	**ARR**	**AS**	**VPR**	**MB**	**SG**	**SP**
Latency	1.00	0.13	0.59	-0.52	-0.42	0.04	-0.10	-0.80	0.24	-0.39	-0.45	-0.39	-0.31	-0.09	0.21	0.05	0.30
Burst Length	0.13	1.00	0.19	0.34	0.24	-0.24	-0.28	-0.11	-0.71	0.26	-0.19	-0.15	0.06	-0.12	-0.54	-0.19	0.13
Frequency	0.59	0.19	1.00	-0.20	-0.12	0.29	-0.18	-0.65	-0.02	-0.31	-0.49	0.10	-0.13	0.00	-0.02	0.13	0.70
Reflectance LI	-0.52	0.34	-0.20	1.00	0.91	0.09	-0.30	0.24	-0.58	0.57	0.45	0.74	0.55	0.26	-0.33	-0.21	0.16
Reflectance LII	-0.42	0.24	-0.12	0.91	1.00	0.42	-0.27	0.13	-0.37	0.42	0.63	0.82	0.54	0.46	-0.14	0.14	0.24
Reflectance LIII	0.04	-0.24	0.29	0.09	0.42	1.00	0.29	-0.05	0.39	-0.10	0.34	0.42	0.23	0.31	0.40	0.65	0.32
Reflectance LV	-0.10	-0.28	-0.18	-0.30	-0.27	0.29	1.00	0.61	0.43	0.14	-0.28	-0.26	0.32	-0.30	0.57	0.04	-0.58
Reflectance LVI	-0.80	-0.11	-0.65	0.24	0.13	-0.05	0.61	1.00	-0.07	0.52	0.12	0.05	0.46	-0.18	0.11	-0.13	-0.65
Deformity	0.24	-0.71	-0.02	-0.58	-0.37	0.39	0.43	-0.07	1.00	-0.63	-0.09	-0.16	-0.32	0.38	0.81	0.61	-0.08
Surface Righting	-0.39	0.26	-0.31	0.57	0.42	-0.10	0.14	0.52	-0.63	1.00	0.19	0.31	0.84	-0.38	-0.18	-0.50	-0.27
Negative Geotaxis	-0.45	-0.19	-0.49	0.45	0.63	0.34	-0.28	0.12	-0.09	0.19	1.00	0.48	0.20	0.31	-0.17	0.23	-0.07
Air Righting	-0.39	-0.15	0.10	0.74	0.82	0.42	-0.26	0.05	-0.16	0.31	0.48	1.00	0.58	0.52	0.06	0.13	0.33
Auditory Startle	-0.31	0.06	-0.13	0.55	0.54	0.23	0.32	0.46	-0.32	0.84	0.20	0.58	1.00	-0.05	0.21	-0.23	-0.27
Visual Placing	-0.09	-0.12	0.00	0.26	0.46	0.31	-0.30	-0.18	0.38	-0.38	0.31	0.52	-0.05	1.00	0.40	0.70	0.27
Marble Burying	0.21	-0.54	-0.02	-0.33	-0.14	0.40	0.57	0.11	0.81	-0.18	-0.17	0.06	0.21	0.40	1.00	0.49	-0.20

Self-grooming	0.05	-0.19	0.13	-0.21	0.14	0.65	0.04	-0.13	0.61	-0.50	0.23	0.13	-0.23	0.70	0.49	1.00	0.29
Social Preference	0.30	0.13	0.70	0.16	0.24	0.32	-0.58	-0.65	-0.08	-0.27	-0.07	0.33	-0.27	0.27	-0.20	0.29	1.00

**6-week-old VPA strong male (N=7)**	**Lat**	**Bh**	**Freq**	**R LI**	**R LII**	**R LIII**	**R LV**	**R LVI**	**Def**	**SRR**	**NGR**	**ARR**	**AS**	**VPR**	**MB**	**SG**	**SP**
Latency	1.00	-0.15	0.43	0.50	0.55	0.61	0.60	0.25	0.01	-0.16	-0.37	0.39	0.64	0.75	0.44	-0.23	0.90
Burst Length	-0.15	1.00	-0.58	-0.49	-0.25	-0.20	-0.10	-0.01	0.14	-0.24	0.30	0.32	0.25	-0.17	-0.57	0.15	-0.17
Frequency	0.43	-0.58	1.00	0.57	0.40	0.32	0.19	-0.18	0.34	-0.38	-0.75	-0.19	0.60	0.24	0.39	-0.26	0.30
Reflectance LI	0.50	-0.49	0.57	1.00	0.94	0.88	0.78	0.44	0.28	-0.51	-0.64	-0.31	0.30	0.46	0.90	-0.71	0.57
Reflectance LII	0.55	-0.25	0.40	0.94	1.00	0.98	0.90	0.51	0.44	-0.60	-0.64	-0.07	0.36	0.60	0.85	-0.60	0.70
Reflectance LIII	0.61	-0.20	0.32	0.88	0.98	1.00	0.97	0.62	0.39	-0.49	-0.53	0.10	0.35	0.74	0.86	-0.55	0.76
Reflectance LV	0.60	-0.10	0.19	0.78	0.90	0.97	1.00	0.77	0.27	-0.33	-0.32	0.22	0.30	0.80	0.85	-0.56	0.72
Reflectance LVI	0.25	-0.01	-0.18	0.44	0.51	0.62	0.77	1.00	-0.22	0.15	0.30	0.09	-0.08	0.55	0.70	-0.64	0.24
Deformity	0.01	0.14	0.34	0.28	0.44	0.39	0.27	-0.22	1.00	-0.76	-0.76	0.23	0.41	0.24	0.09	0.19	0.27
Surface Righting	-0.16	-0.24	-0.38	-0.51	-0.60	-0.49	-0.33	0.15	-0.76	1.00	0.79	0.22	-0.60	0.00	-0.15	0.30	-0.27
Negative Geotaxis	-0.37	0.30	-0.75	-0.64	-0.64	-0.53	-0.32	0.30	-0.76	0.79	1.00	0.10	-0.56	-0.24	-0.34	0.10	-0.50
Air Righting	0.39	0.32	-0.19	-0.31	-0.07	0.10	0.22	0.09	0.23	0.22	0.10	1.00	0.21	0.67	-0.13	0.57	0.48
Auditory Startle	0.64	0.25	0.60	0.30	0.36	0.35	0.30	-0.08	0.41	-0.60	-0.56	0.21	1.00	0.34	0.04	-0.23	0.46
Visual Placing	0.75	-0.17	0.24	0.46	0.60	0.74	0.80	0.55	0.24	0.00	-0.24	0.67	0.34	1.00	0.63	-0.08	0.83
Marble Burying	0.44	-0.57	0.39	0.90	0.85	0.86	0.85	0.70	0.09	-0.15	-0.34	-0.13	0.04	0.63	1.00	-0.65	0.53
Self-grooming	-0.23	0.15	-0.26	-0.71	-0.60	-0.55	-0.56	-0.64	0.19	0.30	0.10	0.57	-0.23	-0.08	-0.65	1.00	-0.08
Social Preference	0.90	-0.17	0.30	0.57	0.70	0.76	0.72	0.24	0.27	-0.27	-0.50	0.48	0.46	0.83	0.53	-0.08	1.00

**3-month-old control male (N=11)**	**Lat**	**Bh**	**Freq**	**R LI**	**R LII**	**R LIII**	**R LV**	**R LVI**	**Def**	**SRR**	**NGR**	**ARR**	**AS**	**VPR**	**MB**	**SG**	**SP**
Latency	1.00	-0.42	0.62	0.23	0.32	0.36	0.46	0.19	--	0.12	0.30	-0.25	-0.10	0.00	-0.05	-0.07	-0.77
Burst Length	-0.42	1.00	-0.37	-0.37	-0.44	-0.52	-0.58	-0.63	--	-0.28	-0.46	0.18	0.42	-0.02	0.23	0.48	0.46
Frequency	0.62	-0.37	1.00	-0.13	-0.05	0.02	0.14	-0.14	--	-0.21	0.26	-0.34	-0.45	-0.10	-0.07	-0.31	-0.40
Reflectance LI	0.23	-0.37	-0.13	1.00	0.99	0.94	0.80	0.53	--	0.53	0.20	0.05	0.19	0.49	-0.04	-0.19	-0.21
Reflectance LII	0.32	-0.44	-0.05	0.99	1.00	0.98	0.88	0.60	--	0.48	0.31	0.01	0.19	0.39	-0.01	-0.20	-0.25
Reflectance LIII	0.36	-0.52	0.02	0.94	0.98	1.00	0.95	0.70	--	0.42	0.44	0.00	0.16	0.31	-0.03	-0.26	-0.25
Reflectance LV	0.46	-0.58	0.14	0.80	0.88	0.95	1.00	0.80	--	0.34	0.59	0.07	0.16	0.18	-0.14	-0.35	-0.30
Reflectance LVI	0.19	-0.63	-0.14	0.53	0.60	0.70	0.80	1.00	--	0.40	0.60	0.29	0.23	0.16	-0.41	-0.54	-0.05
Deformity	--	--	--	--	--	--	--	--	--	--	--	--	--	--	--	--	--
Surface Righting	0.12	-0.28	-0.21	0.53	0.48	0.42	0.34	0.40	--	1.00	-0.24	0.07	0.37	0.74	-0.58	-0.44	-0.01
Negative Geotaxis	0.30	-0.46	0.26	0.20	0.31	0.44	0.59	0.60	--	-0.24	1.00	0.28	-0.14	-0.48	-0.04	-0.47	-0.25
Air Righting	-0.25	0.18	-0.34	0.05	0.01	0.00	0.07	0.29	--	0.07	0.28	1.00	0.38	0.00	-0.61	-0.28	-0.02
Auditory Startle	-0.10	0.42	-0.45	0.19	0.19	0.16	0.16	0.23	--	0.37	-0.14	0.38	1.00	0.21	-0.19	0.13	0.29
Visual Placing	0.00	-0.02	-0.10	0.49	0.39	0.31	0.18	0.16	--	0.74	-0.48	0.00	0.21	1.00	-0.43	-0.31	0.39
Marble Burying	-0.05	0.23	-0.07	-0.04	-0.01	-0.03	-0.14	-0.41	--	-0.58	-0.04	-0.61	-0.19	-0.43	1.00	0.65	0.12
Self-grooming	-0.07	0.48	-0.31	-0.19	-0.20	-0.26	-0.35	-0.54	--	-0.44	-0.47	-0.28	0.13	-0.31	0.65	1.00	-0.15
Social Preference	-0.77	0.46	-0.40	-0.21	-0.25	-0.25	-0.30	-0.05	--	-0.01	-0.25	-0.02	0.29	0.39	0.12	-0.15	1.00

**3-month-old VPA weak male (N=11)**	**Lat**	**Bh**	**Freq**	**R LI**	**R LII**	**R LIII**	**R LV**	**R LVI**	**Def**	**SRR**	**NGR**	**ARR**	**AS**	**VPR**	**MB**	**SG**	**SP**

Latency	1.00	-0.34	-0.20	0.29	0.33	0.40	0.44	0.04	-0.40	0.36	-0.20	0.01	0.14	0.03	-0.16	-0.29	-0.63
Burst Length	-0.34	1.00	-0.38	0.44	0.36	0.12	-0.14	-0.20	-0.32	-0.16	-0.29	-0.45	0.09	-0.34	-0.04	0.65	-0.41
Frequency	-0.20	-0.38	1.00	-0.33	-0.31	-0.21	-0.09	-0.11	0.64	-0.02	0.76	0.48	-0.21	0.27	-0.14	-0.27	-0.27
Reflectance LI	0.29	0.44	-0.33	1.00	0.98	0.86	0.64	-0.12	-0.52	-0.04	-0.41	-0.12	-0.08	-0.32	0.28	0.19	-0.22
Reflectance LII	0.33	0.36	-0.31	0.98	1.00	0.94	0.74	-0.16	-0.47	0.03	-0.37	-0.04	-0.07	-0.24	0.28	0.09	-0.19
Reflectance LIII	0.40	0.12	-0.21	0.86	0.94	1.00	0.91	-0.14	-0.34	0.07	-0.26	0.11	-0.03	-0.03	0.25	-0.17	-0.04
Reflectance LV	0.44	-0.14	-0.09	0.64	0.74	0.91	1.00	0.05	-0.23	-0.05	-0.18	0.17	-0.03	0.21	0.26	-0.32	0.21
Reflectance LVI	0.04	-0.20	-0.11	-0.12	-0.16	-0.14	0.05	1.00	-0.30	-0.24	-0.24	-0.51	-0.59	-0.35	0.40	-0.21	0.41
Deformity	-0.40	-0.32	0.64	-0.52	-0.47	-0.34	-0.23	-0.30	1.00	0.01	0.91	0.69	0.35	0.63	-0.18	-0.48	0.15
Surface Righting	0.36	-0.16	-0.02	-0.04	0.03	0.07	-0.05	-0.24	0.01	1.00	0.24	0.17	-0.05	-0.26	0.08	-0.28	-0.82
Negative Geotaxis	-0.20	-0.29	0.76	-0.41	-0.37	-0.26	-0.18	-0.24	0.91	0.24	1.00	0.67	0.22	0.47	-0.11	-0.53	-0.36
Air Righting	0.01	-0.45	0.48	-0.12	-0.04	0.11	0.17	-0.51	0.69	0.17	0.67	1.00	0.49	0.60	0.12	-0.50	0.10
Auditory Startle	0.14	0.09	-0.21	-0.08	-0.07	-0.03	-0.03	-0.59	0.35	-0.05	0.22	0.49	1.00	0.70	-0.31	-0.06	--
Visual Placing	0.03	-0.34	0.27	-0.32	-0.24	-0.03	0.21	-0.35	0.63	-0.26	0.47	0.60	0.70	1.00	-0.30	-0.32	0.63
Marble Burying	-0.16	-0.04	-0.14	0.28	0.28	0.25	0.26	0.40	-0.18	0.08	-0.11	0.12	-0.31	-0.30	1.00	-0.06	0.79
Self-grooming	-0.29	0.65	-0.27	0.19	0.09	-0.17	-0.32	-0.21	-0.48	-0.28	-0.53	-0.50	-0.06	-0.32	-0.06	1.00	0.93
Social Preference	-0.63	-0.41	-0.27	-0.22	-0.19	-0.04	0.21	0.41	0.15	-0.82	-0.36	0.10	--	0.63	0.79	0.93	1.00

**3-month-old VPA strong male (N=4)**	**Lat**	**Bh**	**Freq**	**R LI**	**R LII**	**R LIII**	**R LV**	**R LVI**	**Def**	**SRR**	**NGR**	**ARR**	**AS**	**VPR**	**MB**	**SG**	**SP**
Latency	1.00	0.21	0.35	-0.46	0.20	0.60	0.28	-0.53	0.98	0.04	-0.21	0.00	-0.98	-0.49	-0.73	-0.18	0.97
Burst Length	0.21	1.00	-0.48	-0.49	-0.39	0.08	-0.18	-0.93	0.16	-0.83	-0.47	-0.50	-0.16	-0.56	0.33	0.63	0.12
Frequency	0.35	-0.48	1.00	-0.53	-0.34	-0.36	-0.46	0.40	0.25	0.88	0.83	-0.35	-0.25	-0.46	-0.88	0.08	0.22
Reflectance LI	-0.46	-0.49	-0.53	1.00	0.78	0.38	0.70	0.46	-0.30	-0.09	-0.42	0.88	0.30	1.00	0.50	-0.75	-0.23
Reflectance LII	0.20	-0.39	-0.34	0.78	1.00	0.84	0.98	0.13	0.37	-0.07	-0.62	0.98	-0.37	0.75	0.04	-0.95	0.43
Reflectance LIII	0.60	0.08	-0.36	0.38	0.84	1.00	0.92	-0.39	0.73	-0.35	-0.80	0.71	-0.73	0.32	-0.10	-0.71	0.77
Reflectance LV	0.28	-0.18	-0.46	0.70	0.98	0.92	1.00	-0.08	0.45	-0.26	-0.77	0.92	-0.45	0.66	0.10	-0.87	0.51
Reflectance LVI	-0.53	-0.93	0.40	0.46	0.13	-0.39	-0.08	1.00	-0.50	0.77	0.61	0.30	0.50	0.54	-0.10	-0.37	-0.47
Deformity	0.98	0.16	0.25	-0.30	0.37	0.73	0.45	-0.50	1.00	0.00	-0.33	0.17	-1.00	-0.33	-0.67	-0.34	1.00
Surface Righting	0.04	-0.83	0.88	-0.09	-0.07	-0.35	-0.26	0.77	0.00	1.00	0.82	0.00	0.00	0.00	-0.70	-0.23	0.00
Negative Geotaxis	-0.21	-0.47	0.83	-0.42	-0.62	-0.80	-0.77	0.61	-0.33	0.82	1.00	-0.52	0.33	-0.33	-0.46	0.35	-0.37
Air Righting	0.00	-0.50	-0.35	0.88	0.98	0.71	0.92	0.30	0.17	0.00	-0.52	1.00	-0.17	0.87	0.13	-0.96	0.24
Auditory Startle	-0.98	-0.16	-0.25	0.30	-0.37	-0.73	-0.45	0.50	-1.00	0.00	0.33	-0.17	1.00	0.33	0.67	0.34	-1.00
Visual Placing	-0.49	-0.56	-0.46	1.00	0.75	0.32	0.66	0.54	-0.33	0.00	-0.33	0.87	0.33	1.00	0.46	-0.75	-0.27
Marble Burying	-0.73	0.33	-0.88	0.50	0.04	-0.10	0.10	-0.10	-0.67	-0.70	-0.46	0.13	0.67	0.46	1.00	0.16	-0.65
Self-grooming	-0.18	0.63	0.08	-0.75	-0.95	-0.71	-0.87	-0.37	-0.34	-0.23	0.35	-0.96	0.34	-0.75	0.16	1.00	-0.40
Social Preference	0.97	0.12	0.22	-0.23	0.43	0.77	0.51	-0.47	1.00	0.00	-0.37	0.24	-1.00	-0.27	-0.65	-0.40	1.00

**6-week-old control female (N=13)**	**Lat**	**Bh**	**Freq**	**R LI**	**R LII**	**R LIII**	**R LV**	**R LVI**	**Def**	**SRR**	**NGR**	**ARR**	**AS**	**VPR**	**MB**	**SG**	**SP**
Latency	1.00	-0.09	-0.40	0.12	0.32	0.47	0.58	0.17	--	-0.15	0.20	-0.05	-0.13	-0.07	-0.01	-0.10	0.36
Burst Length	-0.09	1.00	0.03	0.39	0.55	0.59	0.49	0.31	--	-0.12	-0.26	0.26	0.31	-0.04	0.32	-0.18	-0.12
Frequency	-0.40	0.03	1.00	-0.50	-0.60	-0.63	-0.69	-0.40	--	0.11	0.09	-0.19	-0.19	0.07	0.10	0.56	-0.04
Reflectance LI	0.12	0.39	-0.50	1.00	0.93	0.77	0.51	0.33	--	0.08	0.08	0.33	0.30	0.19	0.32	-0.64	-0.11
Reflectance LII	0.32	0.55	-0.60	0.93	1.00	0.95	0.76	0.50	--	-0.03	-0.04	0.39	0.35	0.18	0.30	-0.60	-0.07

Reflectance LIII	0.47	0.59	-0.63	0.77	0.95	1.00	0.91	0.59	--	-0.15	-0.11	0.39	0.27	0.07	0.26	-0.55	-0.01
Reflectance LV	0.58	0.49	-0.69	0.51	0.76	0.91	1.00	0.74	--	-0.28	-0.16	0.42	0.20	-0.10	0.20	-0.46	0.04
Reflectance LVI	0.17	0.31	-0.40	0.33	0.50	0.59	0.74	1.00	--	-0.45	-0.41	0.21	0.01	-0.22	0.36	-0.26	0.22
Deformity	--	--	--	--	--	--	--	--	--	--	--	--	--	--	--	--	--
Surface Righting	-0.15	-0.12	0.11	0.08	-0.03	-0.15	-0.28	-0.45	--	1.00	0.55	0.45	0.64	0.68	-0.63	-0.17	-0.04
Negative Geotaxis	0.20	-0.26	0.09	0.08	-0.04	-0.11	-0.16	-0.41	--	0.55	1.00	0.56	0.18	0.28	-0.26	0.03	-0.25
Air Righting	-0.05	0.26	-0.19	0.33	0.39	0.39	0.42	0.21	--	0.45	0.56	1.00	0.52	0.32	-0.08	-0.32	-0.52
Auditory Startle	-0.13	0.31	-0.19	0.30	0.35	0.27	0.20	0.01	--	0.64	0.18	0.52	1.00	0.79	-0.57	0.00	-0.19
Visual Placing	-0.07	-0.04	0.07	0.19	0.18	0.07	-0.10	-0.22	--	0.68	0.28	0.32	0.79	1.00	-0.54	0.22	-0.14
Marble Burying	-0.01	0.32	0.10	0.32	0.30	0.26	0.20	0.36	--	-0.63	-0.26	-0.08	-0.57	-0.54	1.00	-0.25	-0.20
Self-grooming	-0.10	-0.18	0.56	-0.64	-0.60	-0.55	-0.46	-0.26	--	-0.17	0.03	-0.32	0.00	0.22	-0.25	1.00	0.04
Social Preference	0.36	-0.12	-0.04	-0.11	-0.07	-0.01	0.04	0.22	--	-0.04	-0.25	-0.52	-0.19	-0.14	-0.20	0.04	1.00

**6-week-old VPA weak female (N=7)**	**Lat**	**Bh**	**Freq**	**R LI**	**R LII**	**R LIII**	**R LV**	**R LVI**	**Def**	**SRR**	**NGR**	**ARR**	**AS**	**VPR**	**MB**	**SG**	**SP**
Latency	1.00	0.52	0.66	0.23	0.26	0.16	0.24	0.33	0.13	-0.19	-0.19	-0.26	0.46	0.13	0.10	0.06	-0.31
Burst Length	0.52	1.00	0.72	-0.06	-0.32	-0.52	-0.60	0.29	0.69	-0.12	0.37	0.45	0.09	-0.07	-0.05	-0.39	-0.83
Frequency	0.66	0.72	1.00	-0.19	-0.30	-0.43	-0.29	0.37	0.34	-0.44	-0.07	0.49	0.32	0.57	0.49	-0.66	-0.41
Reflectance LI	0.23	-0.06	-0.19	1.00	0.94	0.84	0.57	0.51	0.12	-0.02	-0.13	-0.32	0.31	-0.40	-0.44	0.28	-0.06
Reflectance LII	0.26	-0.32	-0.30	0.94	1.00	0.97	0.79	0.35	-0.12	0.01	-0.33	-0.56	0.36	-0.31	-0.37	0.44	0.22
Reflectance LIII	0.16	-0.52	-0.43	0.84	0.97	1.00	0.88	0.23	-0.29	0.10	-0.37	-0.66	0.25	-0.25	-0.28	0.53	0.39
Reflectance LV	0.24	-0.60	-0.29	0.57	0.79	0.88	1.00	0.28	-0.67	0.03	-0.29	-0.66	0.11	0.12	0.14	0.55	0.43
Reflectance LVI	0.33	0.29	0.37	0.51	0.35	0.23	0.28	1.00	-0.12	-0.60	0.31	0.35	0.24	0.26	0.17	-0.20	-0.55
Deformity	0.13	0.69	0.34	0.12	-0.12	-0.29	-0.67	-0.12	1.00	0.05	-0.08	0.34	0.26	-0.42	-0.48	-0.44	-0.36
Surface Righting	-0.19	-0.12	-0.44	-0.02	0.01	0.10	0.03	-0.60	0.05	1.00	0.22	-0.51	-0.71	-0.42	-0.12	0.56	0.18
Negative Geotaxis	-0.19	0.37	-0.07	-0.13	-0.33	-0.37	-0.29	0.31	-0.08	0.22	1.00	0.27	-0.67	-0.17	0.05	0.20	-0.74
Air Righting	-0.26	0.45	0.49	-0.32	-0.56	-0.66	-0.66	0.35	0.34	-0.51	0.27	1.00	0.00	0.41	0.29	-0.87	-0.42
Auditory Startle	0.46	0.09	0.32	0.31	0.36	0.25	0.11	0.24	0.26	-0.71	-0.67	0.00	1.00	0.00	-0.31	-0.30	0.08
Visual Placing	0.13	-0.07	0.57	-0.40	-0.31	-0.25	0.12	0.26	-0.42	-0.42	-0.17	0.41	0.00	1.00	0.94	-0.54	0.20
Marble Burying	0.10	-0.05	0.49	-0.44	-0.37	-0.28	0.14	0.17	-0.48	-0.12	0.05	0.29	-0.31	0.94	1.00	-0.35	0.14
Self-grooming	0.06	-0.39	-0.66	0.28	0.44	0.53	0.55	-0.20	-0.44	0.56	0.20	-0.87	-0.30	-0.54	-0.35	1.00	0.10
Social Preference	-0.31	-0.83	-0.41	-0.06	0.22	0.39	0.43	-0.55	-0.36	0.18	-0.74	-0.42	0.08	0.20	0.14	0.10	1.00

**6-week-old VPA strong female (N=8)**	**Lat**	**Bh**	**Freq**	**R LI**	**R LII**	**R LIII**	**R LV**	**R LVI**	**Def**	**SRR**	**NGR**	**ARR**	**AS**	**VPR**	**MB**	**SG**	**SP**
Latency	1.00	-0.54	0.38	0.24	0.51	0.47	0.38	-0.24	0.16	-0.53	0.04	-0.81	-0.30	0.04	-0.49	0.22	-0.42
Burst Length	-0.54	1.00	-0.47	-0.25	-0.46	-0.29	0.16	0.88	0.20	0.06	0.15	0.43	-0.20	-0.58	0.00	-0.51	0.49
Frequency	0.38	-0.47	1.00	-0.26	-0.03	-0.04	-0.01	-0.43	-0.63	0.15	0.52	-0.07	0.51	0.77	0.12	0.49	0.05
Reflectance LI	0.24	-0.25	-0.26	1.00	0.92	0.86	0.38	-0.11	-0.18	0.35	-0.32	-0.33	0.23	-0.28	0.26	0.19	-0.37
Reflectance LII	0.51	-0.46	-0.03	0.92	1.00	0.97	0.51	-0.26	-0.11	0.22	-0.12	-0.59	0.15	-0.05	0.23	0.24	-0.52
Reflectance LIII	0.47	-0.29	-0.04	0.86	0.97	1.00	0.66	-0.09	-0.07	0.25	0.04	-0.60	0.10	-0.08	0.31	0.16	-0.43
Reflectance LV	0.38	0.16	-0.01	0.38	0.51	0.66	1.00	0.48	0.06	0.14	0.22	-0.67	-0.06	-0.17	0.25	0.05	-0.31
Reflectance LVI	-0.24	0.88	-0.43	-0.11	-0.26	-0.09	0.48	1.00	0.25	-0.06	-0.04	0.01	-0.30	-0.69	-0.06	-0.25	0.41
Deformity	0.16	0.20	-0.63	-0.18	-0.11	-0.07	0.06	0.25	1.00	-0.76	-0.18	-0.34	-0.97	-0.49	-0.55	-0.58	-0.22
Surface Righting	-0.53	0.06	0.15	0.35	0.22	0.25	0.14	-0.06	-0.76	1.00	0.21	0.40	0.88	0.34	0.90	0.24	0.06

Negative Geotaxis	0.04	0.15	0.52	-0.32	-0.12	0.04	0.22	-0.04	-0.18	0.21	1.00	0.14	0.21	0.54	0.20	-0.38	-0.10
Air Righting	-0.81	0.43	-0.07	-0.33	-0.59	-0.60	-0.67	0.01	-0.34	0.40	0.14	1.00	0.40	0.08	0.19	-0.26	0.49
Auditory Startle	-0.30	-0.20	0.51	0.23	0.15	0.10	-0.06	-0.30	-0.97	0.88	0.21	0.40	1.00	0.54	0.69	0.47	0.09
Visual Placing	0.04	-0.58	0.77	-0.28	-0.05	-0.08	-0.17	-0.69	-0.49	0.34	0.54	0.08	0.54	1.00	0.41	0.27	-0.22
Marble Burying	-0.49	0.00	0.12	0.26	0.23	0.31	0.25	-0.06	-0.55	0.90	0.20	0.19	0.69	0.41	1.00	0.31	0.07
Self-grooming	0.22	-0.51	0.49	0.19	0.24	0.16	0.05	-0.25	-0.58	0.24	-0.38	-0.26	0.47	0.27	0.31	1.00	0.27
Social Preference	-0.42	0.49	0.05	-0.37	-0.52	-0.43	-0.31	0.41	-0.22	0.06	-0.10	0.49	0.09	-0.22	0.07	0.27	1.00

**3-month-old control female (N=16)**	**Lat**	**Bh**	**Freq**	**R LI**	**R LII**	**R LIII**	**R LV**	**R LVI**	**Def**	**SRR**	**NGR**	**ARR**	**AS**	**VPR**	**MB**	**SG**	**SP**
Latency	1.00	-0.35	0.04	0.48	0.37	0.22	0.65	0.36	--	-0.16	-0.34	0.19	0.30	-0.46	-0.04	-0.05	-0.31
Burst Length	-0.35	1.00	-0.38	0.00	0.25	0.44	-0.11	-0.17	--	0.05	0.07	0.01	-0.44	-0.03	-0.09	0.22	0.01
Frequency	0.04	-0.38	1.00	-0.20	-0.15	-0.12	-0.16	-0.44	--	-0.02	-0.07	0.01	-0.50	-0.15	-0.03	-0.25	0.44
Reflectance LI	0.48	0.00	-0.20	1.00	0.92	0.73	0.91	0.71	--	0.33	0.26	0.09	-0.09	-0.01	-0.37	-0.54	-0.19
Reflectance LII	0.37	0.25	-0.15	0.92	1.00	0.94	0.88	0.61	--	0.27	0.27	0.09	-0.23	-0.07	-0.42	-0.40	-0.05
Reflectance LIII	0.22	0.44	-0.12	0.73	0.94	1.00	0.74	0.49	--	0.18	0.26	0.11	-0.32	-0.13	-0.40	-0.21	0.01
Reflectance LV	0.65	-0.11	-0.16	0.91	0.88	0.74	1.00	0.72	--	0.16	0.10	0.09	0.08	-0.10	-0.26	-0.26	-0.21
Reflectance LVI	0.36	-0.17	-0.44	0.71	0.61	0.49	0.72	1.00	--	0.27	0.31	0.29	0.08	-0.01	-0.34	-0.12	-0.49
Deformity	--	--	--	--	--	--	--	--	--	--	--	--	--	--	--	--	--
Surface Righting	-0.16	0.05	-0.02	0.33	0.27	0.18	0.16	0.27	--	1.00	0.81	-0.07	-0.02	0.72	-0.45	-0.40	-0.01
Negative Geotaxis	-0.34	0.07	-0.07	0.26	0.27	0.26	0.10	0.31	--	0.81	1.00	-0.01	-0.10	0.61	-0.54	-0.34	-0.18
Air Righting	0.19	0.01	0.01	0.09	0.09	0.11	0.09	0.29	--	-0.07	-0.01	1.00	0.06	-0.58	-0.36	-0.17	0.24
Auditory Startle	0.30	-0.44	-0.50	-0.09	-0.23	-0.32	0.08	0.08	--	-0.02	-0.10	0.06	1.00	0.06	0.25	0.18	-0.16
Visual Placing	-0.46	-0.03	-0.15	-0.01	-0.07	-0.13	-0.10	-0.01	--	0.72	0.61	-0.58	0.06	1.00	-0.10	-0.03	-0.04
Marble Burying	-0.04	-0.09	-0.03	-0.37	-0.42	-0.40	-0.26	-0.34	--	-0.45	-0.54	-0.36	0.25	-0.10	1.00	0.41	-0.36
Self-grooming	-0.05	0.22	-0.25	-0.54	-0.40	-0.21	-0.26	-0.12	--	-0.40	-0.34	-0.17	0.18	-0.03	0.41	1.00	-0.34
Social Preference	-0.31	0.01	0.44	-0.19	-0.05	0.01	-0.21	-0.49	--	-0.01	-0.18	0.24	-0.16	-0.04	-0.36	-0.34	1.00

**3-month-old VPA weak female (N=9)**	**Lat**	**Bh**	**Freq**	**R LI**	**R LII**	**R LIII**	**R LV**	**R LVI**	**Def**	**SRR**	**NGR**	**ARR**	**AS**	**VPR**	**MB**	**SG**	**SP**
Latency	1.00	0.04	0.07	0.44	0.44	0.41	0.39	0.11	0.69	-0.46	0.67	0.30	0.50	0.07	-0.25	-0.56	0.87
Burst Length	0.04	1.00	-0.39	-0.48	-0.55	-0.45	-0.20	-0.21	-0.22	-0.28	0.55	-0.12	0.62	-0.57	0.64	-0.12	-0.31
Frequency	0.07	-0.39	1.00	-0.06	0.01	0.04	-0.02	-0.18	0.11	0.45	-0.31	0.43	-0.47	0.21	-0.34	0.59	0.65
Reflectance LI	0.44	-0.48	-0.06	1.00	0.98	0.86	0.73	0.42	0.81	-0.35	0.24	0.46	0.00	0.81	-0.70	-0.63	0.17
Reflectance LII	0.44	-0.55	0.01	0.98	1.00	0.89	0.71	0.34	0.78	-0.33	0.22	0.47	0.01	0.77	-0.69	-0.62	0.20
Reflectance LIII	0.41	-0.45	0.04	0.86	0.89	1.00	0.91	0.50	0.80	-0.36	0.23	0.48	0.03	0.76	-0.71	-0.59	0.25
Reflectance LV	0.39	-0.20	-0.02	0.73	0.71	0.91	1.00	0.65	0.79	-0.36	0.26	0.46	0.03	0.74	-0.67	-0.51	0.32
Reflectance LVI	0.11	-0.21	-0.18	0.42	0.34	0.50	0.65	1.00	0.54	-0.45	0.10	0.05	-0.09	0.58	-0.53	-0.16	0.23
Deformity	0.69	-0.22	0.11	0.81	0.78	0.80	0.79	0.54	1.00	-0.60	0.50	0.70	0.27	0.71	-0.76	-0.59	0.87
Surface Righting	-0.46	-0.28	0.45	-0.35	-0.33	-0.36	-0.36	-0.45	-0.60	1.00	-0.82	-0.26	-0.81	-0.11	0.13	0.71	0.01
Negative Geotaxis	0.67	0.55	-0.31	0.24	0.22	0.23	0.26	0.10	0.50	-0.82	1.00	0.20	0.90	-0.15	0.17	-0.67	0.10
Air Righting	0.30	-0.12	0.43	0.46	0.47	0.48	0.46	0.05	0.70	-0.26	0.20	1.00	0.12	0.61	-0.70	-0.24	0.55
Auditory Startle	0.50	0.62	-0.47	0.00	0.01	0.03	0.03	-0.09	0.27	-0.81	0.90	0.12	1.00	-0.38	0.32	-0.68	-0.01
Visual Placing	0.07	-0.57	0.21	0.81	0.77	0.76	0.74	0.58	0.71	-0.11	-0.15	0.61	-0.38	1.00	-0.90	-0.21	0.01
Marble Burying	-0.25	0.64	-0.34	-0.70	-0.69	-0.71	-0.67	-0.53	-0.76	0.13	0.17	-0.70	0.32	-0.90	1.00	0.18	-0.44

Self-grooming	-0.56	-0.12	0.59	-0.63	-0.62	-0.59	-0.51	-0.16	-0.59	0.71	-0.67	-0.24	-0.68	-0.21	0.18	1.00	0.07
Social Preference	0.87	-0.31	0.65	0.17	0.20	0.25	0.32	0.23	0.87	0.01	0.10	0.55	-0.01	0.01	-0.44	0.07	1.00

**3-month-old VPA strong female (N=3)**	**Lat**	**Bh**	**Freq**	**R LI**	**R LII**	**R LIII**	**R LV**	**R LVI**	**Def**	**SRR**	**NGR**	**ARR**	**AS**	**VPR**	**MB**	**SG**	**SP**
Latency	1.00	0.89	-0.77	0.81	0.88	0.97	1.00	0.95	-0.34	--	0.34	-0.34	--	-0.34	-0.34	-0.34	-0.34
Burst Length	0.89	1.00	-0.98	0.45	0.56	0.74	0.88	0.70	-0.74	--	0.74	-0.74	--	-0.74	-0.74	-0.74	-0.74
Frequency	-0.77	-0.98	1.00	-0.25	-0.38	-0.58	-0.77	-0.54	0.86	--	-0.86	0.86	--	0.86	0.86	0.86	0.86
Reflectance LI	0.81	0.45	-0.25	1.00	0.99	0.93	0.81	0.95	0.27	--	-0.27	0.27	--	0.27	0.27	0.27	0.27
Reflectance LII	0.88	0.56	-0.38	0.99	1.00	0.97	0.88	0.98	0.14	--	-0.14	0.14	--	0.14	0.14	0.14	0.14
Reflectance LIII	0.97	0.74	-0.58	0.93	0.97	1.00	0.97	1.00	-0.09	--	0.09	-0.09	--	-0.09	-0.09	-0.09	-0.09
Reflectance LV	1.00	0.88	-0.77	0.81	0.88	0.97	1.00	0.95	-0.34	--	0.34	-0.34	--	-0.34	-0.34	-0.34	-0.34
Reflectance LVI	0.95	0.70	-0.54	0.95	0.98	1.00	0.95	1.00	-0.04	--	0.04	-0.04	--	-0.04	-0.04	-0.04	-0.04
Deformity	-0.34	-0.74	0.86	0.27	0.14	-0.09	-0.34	-0.04	1.00	--	-1.00	1.00	--	1.00	1.00	1.00	1.00
Surface Righting	--	--	--	--	--	--	--	--	--	--	--	--	--	--	--	--	--
Negative Geotaxis	0.34	0.74	-0.86	-0.27	-0.14	0.09	0.34	0.04	-1.00	--	1.00	-1.00	--	-1.00	-1.00	-1.00	-1.00
Air Righting	-0.34	-0.74	0.86	0.27	0.14	-0.09	-0.34	-0.04	1.00	--	-1.00	1.00	--	1.00	1.00	1.00	1.00
Auditory Startle	--	--	--	--	--	--	--	--	--	--	--	--	--	--	--	--	--
Visual Placing	-0.34	-0.74	0.86	0.27	0.14	-0.09	-0.34	-0.04	1.00	--	-1.00	1.00	--	1.00	1.00	1.00	1.00
Marble Burying	-0.34	-0.74	0.86	0.27	0.14	-0.09	-0.34	-0.04	1.00	--	-1.00	1.00	--	1.00	1.00	1.00	1.00
Self-grooming	-0.34	-0.74	0.86	0.27	0.14	-0.09	-0.34	-0.04	1.00	--	-1.00	1.00	--	1.00	1.00	1.00	1.00
Social Preference	-0.34	-0.74	0.86	0.27	0.14	-0.09	-0.34	-0.04	1.00	--	-1.00	1.00	--	1.00	1.00	1.00	1.00

Table S2. Pearson correlation values among the morphological, behavioral, and LEC excitability parameters based on MFR-evoked spontaneous electrophysiological activity and IOS recording. The significant values are marked with red, autocorrelations with light gray. The correlation is significant at the 0.05 level. (Lat=latency, Bh=burst length, Freq=frequency, R=reflectance, Def=deformity score, SRR=surface righting reflex, NGR=negative geotaxis reflex, ARR=air righting reflex, AS=auditory startle, VPR=visual placing reflex, MB=marble burying, SG=self-grooming, SP=social preference)

**6-week-old control male (N=14)**	**Lat**	**Bh**	**Freq**	**R LI**	**R LII**	**R LIII**	**R LIV**	**R LV**	**R LVI**	**Def**	**SRR**	**NGR**	**ARR**	**AS**	**VPR**	**MB**	**SG**	**SP**
Latency	1.00	-0.01	-0.04	0.61	0.63	0.70	0.52	0.53	0.30	--	--	--	--	--	--	--	--	--
Burst Length	-0.01	1.00	-0.64	0.12	0.18	0.16	0.29	0.15	-0.35	--	--	--	--	--	--	--	--	--
Frequency	-0.04	-0.64	1.00	0.15	0.06	0.00	-0.15	-0.11	0.26	--	--	--	--	--	--	--	--	--
Reflectance LI	0.61	0.12	0.15	1.00	0.98	0.95	0.79	0.74	0.44	--	--	--	--	--	--	--	--	--
Reflectance LII	0.63	0.18	0.06	0.98	1.00	0.98	0.82	0.74	0.38	--	--	--	--	--	--	--	--	--
Reflectance LIII	0.70	0.16	0.00	0.95	0.98	1.00	0.87	0.80	0.45	--	--	--	--	--	--	--	--	--
Reflectance LIV	0.52	0.29	-0.15	0.79	0.82	0.87	1.00	0.93	0.49	--	--	--	--	--	--	--	--	--
Reflectance LV	0.53	0.15	-0.11	0.74	0.74	0.80	0.93	1.00	0.72	--	--	--	--	--	--	--	--	--
Reflectance LVI	0.30	-0.35	0.26	0.44	0.38	0.45	0.49	0.72	1.00	--	--	--	--	--	--	--	--	--
Deformity	--	--	--	--	--	--	--	--	--	--	--	--	--	--	--	--	--	--
Surface Righting	--	--	--	--	--	--	--	--	--	--	--	--	--	--	--	--	--	--
Negative Geotaxis	--	--	--	--	--	--	--	--	--	--	--	--	--	--	--	--	--	--
Air Righting	--	--	--	--	--	--	--	--	--	--	--	--	--	--	--	--	--	--

Auditory Startle	--	--	--	--	--	--	--	--	--	--	--	--	--	--	--	--	--	--
Visual Placing	--	--	--	--	--	--	--	--	--	--	--	--	--	--	--	--	--	--
Marble Burying	--	--	--	--	--	--	--	--	--	--	--	--	--	--	--	--	--	
Self-grooming	--	--	--	--	--	--	--	--	--	--	--	--	--	--	--	--	--	
Social Preference	--	--	--	--	--	--	--	--	--	--	--	--	--	--	--	--	--	--

**6-week-old VPA weak male (N=10)**	**Lat**	**Bh**	**Freq**	**R LI**	**R LII**	**R LIII**	**R LIV**	**R LV**	**R LVI**	**Def**	**SRR**	**NGR**	**ARR**	**AS**	**VPR**	**MB**	**SG**	**SP**
Latency	1.00	0.29	-0.28	0.53	0.59	0.61	0.62	0.48	0.18	0.25	0.08	-0.01	-0.28	0.42	-0.22	0.24	0.52	0.52
Burst Length	0.29	1.00	-0.24	0.36	0.47	0.54	0.49	0.43	-0.21	0.10	0.33	0.27	-0.14	-0.15	0.72	0.24	0.26	0.19
Frequency	-0.28	-0.24	1.00	-0.28	-0.20	-0.09	-0.01	0.28	0.04	0.08	-0.03	-0.59	0.05	-0.05	0.22	0.50	0.14	-0.03
Reflectance LI	0.53	0.36	-0.28	1.00	0.97	0.86	0.81	0.71	0.40	-0.36	0.01	0.47	-0.03	0.28	0.08	0.09	0.44	0.18
Reflectance LII	0.59	0.47	-0.20	0.97	1.00	0.95	0.92	0.82	0.37	-0.20	0.09	0.31	-0.07	0.26	0.17	0.29	0.52	0.13
Reflectance LIII	0.61	0.54	-0.09	0.86	0.95	1.00	0.99	0.92	0.44	0.02	0.10	0.14	-0.17	0.17	0.20	0.49	0.47	0.09
Reflectance LIV	0.62	0.49	-0.01	0.81	0.92	0.99	1.00	0.93	0.46	0.15	0.02	-0.01	-0.17	0.24	0.20	0.61	0.54	0.01
Reflectance LV	0.48	0.43	0.28	0.71	0.82	0.92	0.93	1.00	0.52	0.04	0.07	-0.04	-0.13	0.13	0.22	0.64	0.50	0.11
Reflectance LVI	0.18	-0.21	0.04	0.40	0.37	0.44	0.46	0.52	1.00	0.02	-0.43	0.04	-0.19	-0.02	-0.49	0.27	0.07	-0.16
Deformity	0.25	0.10	0.08	-0.36	-0.20	0.02	0.15	0.04	0.02	1.00	-0.24	-0.77	-0.26	0.18	0.00	0.70	0.21	-0.37
Surface Righting	0.08	0.33	-0.03	0.01	0.09	0.10	0.02	0.07	-0.43	-0.24	1.00	0.28	0.57	0.14	0.18	-0.17	-0.12	0.41
Negative Geotaxis	-0.01	0.27	-0.59	0.47	0.31	0.14	-0.01	-0.04	0.04	-0.77	0.28	1.00	0.23	-0.14	0.00	-0.72	-0.28	0.39
Air Righting	-0.28	-0.14	0.05	-0.03	-0.07	-0.17	-0.17	-0.13	-0.19	-0.26	0.57	0.23	1.00	0.57	0.00	-0.17	-0.14	-0.03
Auditory Startle	0.42	-0.15	-0.05	0.28	0.26	0.17	0.24	0.13	-0.02	0.18	0.14	-0.14	0.57	1.00	-0.19	0.22	0.50	0.01
Visual Placing	-0.22	0.72	0.22	0.08	0.17	0.20	0.20	0.22	-0.49	0.00	0.18	0.00	0.00	-0.19	1.00	0.29	0.21	-0.09
Marble Burying	0.24	0.24	0.50	0.09	0.29	0.49	0.61	0.64	0.27	0.70	-0.17	-0.72	-0.17	0.22	0.29	1.00	0.51	-0.36
Self-grooming	0.52	0.26	0.14	0.44	0.52	0.47	0.54	0.50	0.07	0.21	-0.12	-0.28	-0.14	0.50	0.21	0.51	1.00	-0.01
Social Preference	0.52	0.19	-0.03	0.18	0.13	0.09	0.01	0.11	-0.16	-0.37	0.41	0.39	-0.03	0.01	-0.09	-0.36	-0.01	1.00

**6-week-old VPA strong male (N=5)**	**Lat**	**Bh**	**Freq**	**R LI**	**R LII**	**R LIII**	**R LIV**	**R LV**	**R LVI**	**Def**	**SRR**	**NGR**	**ARR**	**AS**	**VPR**	**MB**	**SG**	**SP**
Latency	1.00	0.05	0.76	0.42	0.50	0.53	0.49	0.29	-0.36	-0.39	-0.49	-0.47	-0.35	0.71	-0.06	0.08	-0.85	0.47
Burst Length	0.05	1.00	-0.47	-0.58	-0.54	-0.41	-0.35	-0.37	-0.39	-0.11	0.67	0.85	0.90	-0.64	0.91	0.87	0.37	0.73
Frequency	0.76	-0.47	1.00	0.77	0.73	0.50	0.39	0.19	-0.37	-0.13	-0.51	-0.77	-0.71	0.81	-0.36	-0.29	-0.89	0.10
Reflectance LI	0.42	-0.58	0.77	1.00	0.98	0.77	0.69	0.57	0.13	-0.51	-0.38	-0.66	-0.59	0.75	-0.32	-0.68	-0.82	-0.42
Reflectance LII	0.50	-0.54	0.73	0.98	1.00	0.89	0.83	0.72	0.24	-0.65	-0.48	-0.67	-0.58	0.82	-0.34	-0.70	-0.87	-0.42
Reflectance LIII	0.53	-0.41	0.50	0.77	0.89	1.00	0.99	0.94	0.51	-0.83	-0.63	-0.60	-0.48	0.82	-0.38	-0.69	-0.81	-0.44
Reflectance LIV	0.49	-0.35	0.39	0.69	0.83	0.99	1.00	0.97	0.58	-0.87	-0.62	-0.53	-0.41	0.77	-0.35	-0.67	-0.75	-0.45
Reflectance LV	0.29	-0.37	0.19	0.57	0.72	0.94	0.97	1.00	0.76	-0.84	-0.60	-0.46	-0.35	0.66	-0.40	-0.73	-0.57	-0.59
Reflectance LVI	-0.36	-0.39	-0.37	0.13	0.24	0.51	0.58	0.76	1.00	-0.47	-0.38	-0.17	-0.16	0.19	-0.44	-0.72	0.05	-0.84
Deformity	-0.39	-0.11	-0.13	-0.51	-0.65	-0.83	-0.87	-0.84	-0.47	1.00	0.18	0.06	-0.09	-0.41	-0.15	0.34	0.54	0.23
Surface Righting	-0.49	0.67	-0.51	-0.38	-0.48	-0.63	-0.62	-0.60	-0.38	0.18	1.00	0.88	0.85	-0.87	0.87	0.62	0.63	0.33
Negative Geotaxis	-0.47	0.85	-0.77	-0.66	-0.67	-0.60	-0.53	-0.46	-0.17	0.06	0.88	1.00	0.99	-0.92	0.87	0.70	0.74	0.38
Air Righting	-0.35	0.90	-0.71	-0.59	-0.58	-0.48	-0.41	-0.35	-0.16	-0.09	0.85	0.99	1.00	-0.84	0.91	0.70	0.62	0.42
Auditory Startle	0.71	-0.64	0.81	0.75	0.82	0.82	0.77	0.66	0.19	-0.41	-0.87	-0.92	-0.84	1.00	-0.67	-0.63	-0.92	-0.26
Visual Placing	-0.06	0.91	-0.36	-0.32	-0.34	-0.38	-0.35	-0.40	-0.44	-0.15	0.87	0.87	0.91	-0.67	1.00	0.75	0.33	0.59
Marble Burying	0.08	0.87	-0.29	-0.68	-0.70	-0.69	-0.67	-0.73	-0.72	0.34	0.62	0.70	0.70	-0.63	0.75	1.00	0.41	0.91
Self-grooming	-0.85	0.37	-0.89	-0.82	-0.87	-0.81	-0.75	-0.57	0.05	0.54	0.63	0.74	0.62	-0.92	0.33	0.41	1.00	0.02

Social Preference	0.47	0.73	0.10	-0.42	-0.42	-0.44	-0.45	-0.59	-0.84	0.23	0.33	0.38	0.42	-0.26	0.59	0.91	0.02	1.00

**3-month-old control male (N=10)**	**Lat**	**Bh**	**Freq**	**R LI**	**R LII**	**R LIII**	**R LIV**	**R LV**	**R LVI**	**Def**	**SRR**	**NGR**	**ARR**	**AS**	**VPR**	**MB**	**SG**	**SP**
Latency	1.00	-0.15	0.35	0.23	0.26	0.29	0.27	0.39	0.35	--	0.42	0.24	-0.41	0.10	0.09	0.83	0.53	-0.84
Burst Length	-0.15	1.00	-0.45	-0.60	-0.58	-0.58	-0.64	-0.61	-0.61	--	-0.72	-0.83	-0.65	-0.79	-0.91	0.35	0.71	0.62
Frequency	0.35	-0.45	1.00	0.11	0.06	0.14	0.28	0.28	0.09	--	-0.08	0.08	0.65	0.52	0.65	-0.41	-0.89	1.00
Reflectance LI	0.23	-0.60	0.11	1.00	0.99	0.95	0.91	0.91	0.75	--	0.85	0.60	0.36	0.40	0.64	-0.22	-0.43	-0.98
Reflectance LII	0.26	-0.58	0.06	0.99	1.00	0.97	0.91	0.90	0.71	--	0.80	0.63	0.40	0.50	0.72	-0.17	-0.43	-0.99
Reflectance LIII	0.29	-0.58	0.14	0.95	0.97	1.00	0.96	0.93	0.63	--	0.79	0.75	0.46	0.64	0.82	-0.17	-0.48	-1.00
Reflectance LIV	0.27	-0.64	0.28	0.91	0.91	0.96	1.00	0.97	0.66	--	0.79	0.83	0.39	0.63	0.81	-0.30	-0.47	-0.81
Reflectance LV	0.39	-0.61	0.28	0.91	0.90	0.93	0.97	1.00	0.74	--	0.85	0.79	0.23	0.51	0.71	-0.20	-0.33	-0.93
Reflectance LVI	0.35	-0.61	0.09	0.75	0.71	0.63	0.66	0.74	1.00	--	0.88	0.64	-0.13	0.20	0.40	-0.02	-0.01	-0.98
Deformity	--	--	--	--	--	--	--	--	--	--	--	--	--	--	--	--	--	--
Surface Righting	0.42	-0.72	-0.08	0.85	0.80	0.79	0.79	0.85	0.88	--	1.00	0.64	0.10	0.26	0.43	-0.01	-0.31	-0.93
Negative Geotaxis	0.24	-0.83	0.08	0.60	0.63	0.75	0.83	0.79	0.64	--	0.64	1.00	0.27	0.77	0.79	-0.27	-0.41	-0.63
Air Righting	-0.41	-0.65	0.65	0.36	0.40	0.46	0.39	0.23	-0.13	--	0.10	0.27	1.00	0.68	0.72	-0.41	-0.96	0.99
Auditory Startle	0.10	-0.79	0.52	0.40	0.50	0.64	0.63	0.51	0.20	--	0.26	0.77	0.68	1.00	0.95	-0.15	-0.59	--
Visual Placing	0.09	-0.91	0.65	0.64	0.72	0.82	0.81	0.71	0.40	--	0.43	0.79	0.72	0.95	1.00	-0.25	-0.66	--
Marble Burying	0.83	0.35	-0.41	-0.22	-0.17	-0.17	-0.30	-0.20	-0.02	--	-0.01	-0.27	-0.41	-0.15	-0.25	1.00	0.61	-1.00
Self-grooming	0.53	0.71	-0.89	-0.43	-0.43	-0.48	-0.47	-0.33	-0.01	--	-0.31	-0.41	-0.96	-0.59	-0.66	0.61	1.00	-1.00
Social Preference	-0.84	0.62	1.00	-0.98	-0.99	-1.00	-0.81	-0.93	-0.98	--	-0.93	-0.63	0.99	--	--	-1.00	-1.00	1.00

**3-month-old VPA weak male (N=13)**	**Lat**	**Bh**	**Freq**	**R LI**	**R LII**	**R LIII**	**R LIV**	**R LV**	**R LVI**	**Def**	**SRR**	**NGR**	**ARR**	**AS**	**VPR**	**MB**	**SG**	**SP**
Latency	1.00	-0.55	0.16	0.73	0.66	0.68	0.67	0.60	0.55	0.26	-0.35	-0.02	-0.34	-0.15	-0.17	0.18	-0.34	-0.89
Burst Length	-0.55	1.00	-0.51	-0.74	-0.70	-0.70	-0.66	-0.54	-0.35	-0.42	0.60	-0.03	0.35	0.14	0.06	0.39	-0.07	0.34
Frequency	0.16	-0.51	1.00	0.41	0.43	0.38	0.28	0.22	0.38	0.15	-0.41	0.21	-0.49	-0.09	0.41	-0.35	0.10	0.13
Reflectance LI	0.73	-0.74	0.41	1.00	0.99	0.98	0.94	0.83	0.36	0.36	-0.43	-0.09	-0.32	-0.24	-0.21	-0.14	-0.08	-0.60
Reflectance LII	0.66	-0.70	0.43	0.99	1.00	0.99	0.92	0.79	0.25	0.41	-0.34	-0.05	-0.29	-0.24	-0.15	-0.14	-0.09	-0.50
Reflectance LIII	0.68	-0.70	0.38	0.98	0.99	1.00	0.97	0.85	0.28	0.40	-0.37	-0.10	-0.22	-0.32	-0.20	-0.17	0.02	-0.53
Reflectance LIV	0.67	-0.66	0.28	0.94	0.92	0.97	1.00	0.94	0.37	0.30	-0.44	-0.25	-0.16	-0.41	-0.29	-0.19	0.18	-0.59
Reflectance LV	0.60	-0.54	0.22	0.83	0.79	0.85	0.94	1.00	0.55	0.12	-0.53	-0.39	-0.12	-0.39	-0.28	-0.13	0.30	-0.60
Reflectance LVI	0.55	-0.35	0.38	0.36	0.25	0.28	0.37	0.55	1.00	-0.23	-0.65	-0.35	-0.23	0.00	-0.03	0.07	0.12	-0.52
Deformity	0.26	-0.42	0.15	0.36	0.41	0.40	0.30	0.12	-0.23	1.00	-0.05	0.46	-0.23	0.14	0.49	-0.09	-0.28	0.15
Surface Righting	-0.35	0.60	-0.41	-0.43	-0.34	-0.37	-0.44	-0.53	-0.65	-0.05	1.00	0.06	0.43	-0.13	0.09	0.28	-0.07	0.50
Negative Geotaxis	-0.02	-0.03	0.21	-0.09	-0.05	-0.10	-0.25	-0.39	-0.35	0.46	0.06	1.00	-0.13	0.12	0.57	-0.05	-0.43	0.22
Air Righting	-0.34	0.35	-0.49	-0.32	-0.29	-0.22	-0.16	-0.12	-0.23	-0.23	0.43	-0.13	1.00	-0.22	-0.17	0.21	0.47	0.44
Auditory Startle	-0.15	0.14	-0.09	-0.24	-0.24	-0.32	-0.41	-0.39	0.00	0.14	-0.13	0.12	-0.22	1.00	0.28	0.51	-0.67	0.19
Visual Placing	-0.17	0.06	0.41	-0.21	-0.15	-0.20	-0.29	-0.28	-0.03	0.49	0.09	0.57	-0.17	0.28	1.00	0.20	-0.22	0.79
Marble Burying	0.18	0.39	-0.35	-0.14	-0.14	-0.17	-0.19	-0.13	0.07	-0.09	0.28	-0.05	0.21	0.51	0.20	1.00	-0.57	0.07
Self-grooming	-0.34	-0.07	0.10	-0.08	-0.09	0.02	0.18	0.30	0.12	-0.28	-0.07	-0.43	0.47	-0.67	-0.22	-0.57	1.00	0.27
Social Preference	-0.89	0.34	0.13	-0.60	-0.50	-0.53	-0.59	-0.60	-0.52	0.15	0.50	0.22	0.44	0.19	0.79	0.07	0.27	1.00

**3-month-old VPA strong male (N=7)**	**Lat**	**Bh**	**Freq**	**R LI**	**R LII**	**R LIII**	**R LIV**	**R LV**	**R LVI**	**Def**	**SRR**	**NGR**	**ARR**	**AS**	**VPR**	**MB**	**SG**	**SP**

Latency	1.00	0.93	0.29	0.46	0.57	0.62	0.67	0.55	-0.49	-0.72	-0.52	-0.69	-0.69	-0.85	-0.64	-0.68	-0.39	0.69
Burst Length	0.93	1.00	0.03	0.36	0.49	0.56	0.60	0.42	-0.59	-0.69	-0.60	-0.82	-0.62	-0.90	-0.79	-0.69	-0.30	0.68
Frequency	0.29	0.03	1.00	0.48	0.50	0.46	0.53	0.57	0.15	-0.10	-0.20	-0.02	-0.24	-0.01	0.07	0.16	-0.26	-0.10
Reflectance LI	0.46	0.36	0.48	1.00	0.98	0.87	0.69	0.57	0.22	-0.76	-0.51	-0.42	-0.89	-0.15	-0.44	0.21	0.42	-0.18
Reflectance LII	0.57	0.49	0.50	0.98	1.00	0.94	0.81	0.69	0.17	-0.80	-0.62	-0.56	-0.92	-0.27	-0.58	0.11	0.35	-0.08
Reflectance LIII	0.62	0.56	0.46	0.87	0.94	1.00	0.95	0.86	0.23	-0.80	-0.74	-0.72	-0.89	-0.32	-0.74	0.09	0.37	-0.08
Reflectance LIV	0.67	0.60	0.53	0.69	0.81	0.95	1.00	0.96	0.17	-0.69	-0.75	-0.75	-0.76	-0.39	-0.74	0.01	0.20	0.00
Reflectance LV	0.55	0.42	0.57	0.57	0.69	0.86	0.96	1.00	0.35	-0.57	-0.61	-0.59	-0.64	-0.21	-0.59	0.15	0.24	-0.13
Reflectance LVI	-0.49	-0.59	0.15	0.22	0.17	0.23	0.17	0.35	1.00	-0.05	0.17	0.31	-0.11	0.81	0.20	0.93	0.72	-0.93
Deformity	-0.72	-0.69	-0.10	-0.76	-0.80	-0.80	-0.69	-0.57	-0.05	1.00	0.38	0.55	0.96	0.37	0.61	0.09	-0.23	-0.07
Surface Righting	-0.52	-0.60	-0.20	-0.51	-0.62	-0.74	-0.75	-0.61	0.17	0.38	1.00	0.92	0.51	0.60	0.89	0.30	-0.30	-0.32
Negative Geotaxis	-0.69	-0.82	-0.02	-0.42	-0.56	-0.72	-0.75	-0.59	0.31	0.55	0.92	1.00	0.58	0.75	0.99	0.44	-0.14	-0.43
Air Righting	-0.69	-0.62	-0.24	-0.89	-0.92	-0.89	-0.76	-0.64	-0.11	0.96	0.51	0.58	1.00	0.35	0.62	0.05	-0.37	-0.04
Auditory Startle	-0.85	-0.90	-0.01	-0.15	-0.27	-0.32	-0.39	-0.21	0.81	0.37	0.60	0.75	0.35	1.00	0.66	0.90	0.43	-0.91
Visual Placing	-0.64	-0.79	0.07	-0.44	-0.58	-0.74	-0.74	-0.59	0.20	0.61	0.89	0.99	0.62	0.66	1.00	0.34	-0.27	-0.32
Marble Burying	-0.68	-0.69	0.16	0.21	0.11	0.09	0.01	0.15	0.93	0.09	0.30	0.44	0.05	0.90	0.34	1.00	0.58	-1.00
Self-grooming	-0.39	-0.30	-0.26	0.42	0.35	0.37	0.20	0.24	0.72	-0.23	-0.30	-0.14	-0.37	0.43	-0.27	0.58	1.00	-0.60
Social Preference	0.69	0.68	-0.10	-0.18	-0.08	-0.08	0.00	-0.13	-0.93	-0.07	-0.32	-0.43	-0.04	-0.91	-0.32	-1.00	-0.60	1.00

**6-week-old control female (N=15)**	**Lat**	**Bh**	**Freq**	**R LI**	**R LII**	**R LIII**	**R LIV**	**R LV**	**R LVI**	**Def**	**SRR**	**NGR**	**ARR**	**AS**	**VPR**	**MB**	**SG**	**SP**
Latency	1.00	0.29	-0.18	0.12	0.11	0.10	0.12	0.05	-0.15	--	0.26	0.42	-0.69	-0.48	-0.34	0.46	-0.14	-0.04
Burst Length	0.29	1.00	-0.10	-0.27	-0.32	-0.32	-0.28	-0.26	-0.39	--	-0.32	0.25	0.07	0.06	0.08	-0.13	-0.21	-0.74
Frequency	-0.18	-0.10	1.00	0.25	0.30	0.31	0.26	0.32	0.32	--	0.01	-0.24	0.28	-0.11	-0.20	0.43	0.55	0.14
Reflectance LI	0.12	-0.27	0.25	1.00	0.98	0.94	0.92	0.91	0.59	--	0.84	-0.02	-0.52	0.21	0.09	0.22	0.53	0.17
Reflectance LII	0.11	-0.32	0.30	0.98	1.00	0.98	0.96	0.94	0.62	--	0.76	-0.13	-0.44	0.11	-0.02	0.31	0.49	0.23
Reflectance LIII	0.10	-0.32	0.31	0.94	0.98	1.00	0.99	0.97	0.70	--	0.76	-0.28	-0.37	0.14	-0.02	0.25	0.40	0.38
Reflectance LIV	0.12	-0.28	0.26	0.92	0.96	0.99	1.00	0.98	0.68	--	0.75	-0.22	-0.40	0.10	-0.07	0.26	0.34	0.30
Reflectance LV	0.05	-0.26	0.32	0.91	0.94	0.97	0.98	1.00	0.77	--	0.76	-0.26	-0.39	0.15	-0.02	0.20	0.30	0.36
Reflectance LVI	-0.15	-0.39	0.32	0.59	0.62	0.70	0.68	0.77	1.00	--	0.66	-0.46	-0.30	0.44	0.44	-0.18	0.16	0.73
Deformity	--	--	--	--	--	--	--	--	--	--	--	--	--	--	--	--	--	--
Surface Righting	0.26	-0.32	0.01	0.84	0.76	0.76	0.75	0.76	0.66	--	1.00	0.00	-0.56	0.57	0.45	-0.26	0.40	0.32
Negative Geotaxis	0.42	0.25	-0.24	-0.02	-0.13	-0.28	-0.22	-0.26	-0.46	--	0.00	1.00	-0.34	-0.13	0.00	0.04	0.37	-0.74
Air Righting	-0.69	0.07	0.28	-0.52	-0.44	-0.37	-0.40	-0.39	-0.30	--	-0.56	-0.34	1.00	0.00	-0.28	-0.23	0.03	0.06
Auditory Startle	-0.48	0.06	-0.11	0.21	0.11	0.14	0.10	0.15	0.44	--	0.57	-0.13	0.00	1.00	0.87	-0.79	0.37	0.06
Visual Placing	-0.34	0.08	-0.20	0.09	-0.02	-0.02	-0.07	-0.02	0.44	--	0.45	0.00	-0.28	0.87	1.00	-0.58	0.32	-0.03
Marble Burying	0.46	-0.13	0.43	0.22	0.31	0.25	0.26	0.20	-0.18	--	-0.26	0.04	-0.23	-0.79	-0.58	1.00	0.05	-0.08
Self-grooming	-0.14	-0.21	0.55	0.53	0.49	0.40	0.34	0.30	0.16	--	0.40	0.37	0.03	0.37	0.32	0.05	1.00	-0.26
Social Preference	-0.04	-0.74	0.14	0.17	0.23	0.38	0.30	0.36	0.73	--	0.32	-0.74	0.06	0.06	-0.03	-0.08	-0.26	1.00

**6-week-old VPA weak female (N=5)**	**Lat**	**Bh**	**Freq**	**R LI**	**R LII**	**R LIII**	**R LIV**	**R LV**	**R LVI**	**Def**	**SRR**	**NGR**	**ARR**	**AS**	**VPR**	**MB**	**SG**	**SP**
Latency	1.00	0.40	0.10	-0.85	-0.77	-0.69	-0.59	-0.80	-0.66	0.01	0.41	-0.75	-0.13	0.12	-0.50	-0.45	0.27	0.94
Burst Length	0.40	1.00	-0.70	-0.70	-0.82	-0.89	-0.89	-0.66	-0.74	0.57	-0.27	-0.84	-0.89	-0.84	-0.48	0.43	-0.05	0.19
Frequency	0.10	-0.70	1.00	0.03	0.21	0.35	0.33	-0.04	0.25	-0.65	0.32	0.28	0.81	0.67	-0.04	-0.92	-0.34	0.40
Reflectance LI	-0.85	-0.70	0.03	1.00	0.98	0.94	0.91	0.97	0.79	-0.21	-0.16	0.95	0.44	0.35	0.73	0.33	0.16	-0.81

Reflectance LII	-0.77	-0.82	0.21	0.98	1.00	0.98	0.96	0.95	0.82	-0.32	-0.05	0.98	0.59	0.51	0.71	0.15	0.16	-0.69
Reflectance LIII	-0.69	-0.89	0.35	0.94	0.98	1.00	0.99	0.88	0.77	-0.48	-0.04	0.99	0.72	0.62	0.73	0.00	0.17	-0.58
Reflectance LIV	-0.59	-0.89	0.33	0.91	0.96	0.99	1.00	0.85	0.72	-0.51	0.00	0.97	0.75	0.70	0.77	0.00	0.31	-0.51
Reflectance LV	-0.80	-0.66	-0.04	0.97	0.95	0.88	0.85	1.00	0.88	-0.01	0.04	0.88	0.32	0.34	0.58	0.37	0.16	-0.77
Reflectance LVI	-0.66	-0.74	0.25	0.79	0.82	0.77	0.72	0.88	1.00	0.10	0.40	0.73	0.34	0.41	0.20	0.03	-0.15	-0.51
Deformity	0.01	0.57	-0.65	-0.21	-0.32	-0.48	-0.51	-0.01	0.10	1.00	0.33	-0.48	-0.87	-0.61	-0.61	0.51	-0.19	-0.10
Surface Righting	0.41	-0.27	0.32	-0.16	-0.05	-0.04	0.00	0.04	0.40	0.33	1.00	-0.16	0.07	0.47	-0.53	-0.44	0.00	0.52
Negative Geotaxis	-0.75	-0.84	0.28	0.95	0.98	0.99	0.97	0.88	0.73	-0.48	-0.16	1.00	0.68	0.54	0.78	0.08	0.16	-0.66
Air Righting	-0.13	-0.89	0.81	0.44	0.59	0.72	0.75	0.32	0.34	-0.87	0.07	0.68	1.00	0.87	0.53	-0.61	0.14	0.06
Auditory Startle	0.12	-0.84	0.67	0.35	0.51	0.62	0.70	0.34	0.41	-0.61	0.47	0.54	0.87	1.00	0.38	-0.57	0.40	0.26
Visual Placing	-0.50	-0.48	-0.04	0.73	0.71	0.73	0.77	0.58	0.20	-0.61	-0.53	0.78	0.53	0.38	1.00	0.31	0.56	-0.58
Marble Burying	-0.45	0.43	-0.92	0.33	0.15	0.00	0.00	0.37	0.03	0.51	-0.44	0.08	-0.61	-0.57	0.31	1.00	0.31	-0.71
Self-grooming	0.27	-0.05	-0.34	0.16	0.16	0.17	0.31	0.16	-0.15	-0.19	0.00	0.16	0.14	0.40	0.56	0.31	1.00	0.03
Social Preference	0.94	0.19	0.40	-0.81	-0.69	-0.58	-0.51	-0.77	-0.51	-0.10	0.52	-0.66	0.06	0.26	-0.58	-0.71	0.03	1.00

**6-week-old VPA strong female (N=6)**	**Lat**	**Bh**	**Freq**	**R LI**	**R LII**	**R LIII**	**R LIV**	**R LV**	**R LVI**	**Def**	**SRR**	**NGR**	**ARR**	**AS**	**VPR**	**MB**	**SG**	**SP**
Latency	1.00	0.91	0.84	-0.08	-0.12	-0.04	0.07	0.16	0.07	-0.02	-0.07	0.14	-0.18	0.24	0.44	0.18	-0.12	-0.51
Burst Length	0.91	1.00	0.72	-0.31	-0.36	-0.32	-0.26	-0.16	-0.03	-0.03	-0.23	0.23	-0.13	0.55	0.19	0.24	0.06	-0.34
Frequency	0.84	0.72	1.00	-0.24	-0.19	-0.11	-0.04	-0.01	-0.25	0.31	-0.31	-0.05	0.31	0.12	0.06	0.36	-0.59	-0.57
Reflectance LI	-0.08	-0.31	-0.24	1.00	0.98	0.96	0.91	0.92	0.76	-0.61	0.60	0.61	-0.36	-0.36	0.33	0.00	-0.11	0.36
Reflectance LII	-0.12	-0.36	-0.19	0.98	1.00	0.99	0.94	0.93	0.76	-0.43	0.66	0.56	-0.17	-0.50	0.22	0.16	-0.23	0.25
Reflectance LIII	-0.04	-0.32	-0.11	0.96	0.99	1.00	0.97	0.96	0.76	-0.39	0.68	0.49	-0.18	-0.55	0.31	0.14	-0.24	0.15
Reflectance LIV	0.07	-0.26	-0.04	0.91	0.94	0.97	1.00	0.99	0.76	-0.31	0.74	0.35	-0.24	-0.65	0.47	0.09	-0.18	-0.04
Reflectance LV	0.16	-0.16	-0.01	0.92	0.93	0.96	0.99	1.00	0.79	-0.38	0.73	0.41	-0.31	-0.57	0.52	0.09	-0.14	-0.03
Reflectance LVI	0.07	-0.03	-0.25	0.76	0.76	0.76	0.76	0.79	1.00	-0.35	0.90	0.64	-0.31	-0.36	0.29	0.33	0.28	0.04
Deformity	-0.02	-0.03	0.31	-0.61	-0.43	-0.39	-0.31	-0.38	-0.35	1.00	0.00	-0.61	0.77	-0.42	-0.39	0.48	-0.26	-0.74
Surface Righting	-0.07	-0.23	-0.31	0.60	0.66	0.68	0.74	0.73	0.90	0.00	1.00	0.25	-0.16	-0.69	0.32	0.31	0.31	-0.24
Negative Geotaxis	0.14	0.23	-0.05	0.61	0.56	0.49	0.35	0.41	0.64	-0.61	0.25	1.00	-0.16	0.34	-0.16	0.39	-0.02	0.54
Air Righting	-0.18	-0.13	0.31	-0.36	-0.17	-0.18	-0.24	-0.31	-0.31	0.77	-0.16	-0.16	1.00	-0.22	-0.80	0.71	-0.64	-0.29
Auditory Startle	0.24	0.55	0.12	-0.36	-0.50	-0.55	-0.65	-0.57	-0.36	-0.42	-0.69	0.34	-0.22	1.00	-0.22	-0.15	0.17	0.48
Visual Placing	0.44	0.19	0.06	0.33	0.22	0.31	0.47	0.52	0.29	-0.39	0.32	-0.16	-0.80	-0.22	1.00	-0.57	0.37	-0.26
Marble Burying	0.18	0.24	0.36	0.00	0.16	0.14	0.09	0.09	0.33	0.48	0.31	0.39	0.71	-0.15	-0.57	1.00	-0.37	-0.31
Self-grooming	-0.12	0.06	-0.59	-0.11	-0.23	-0.24	-0.18	-0.14	0.28	-0.26	0.31	-0.02	-0.64	0.17	0.37	-0.37	1.00	0.09
Social Preference	-0.51	-0.34	-0.57	0.36	0.25	0.15	-0.04	-0.03	0.04	-0.74	-0.24	0.54	-0.29	0.48	-0.26	-0.31	0.09	1.00

**3-month-old control female (N=13)**	**Lat**	**Bh**	**Freq**	**R LI**	**R LII**	**R LIII**	**R LIV**	**R LV**	**R LVI**	**Def**	**SRR**	**NGR**	**ARR**	**AS**	**VPR**	**MB**	**SG**	**SP**
Latency	1.00	0.54	-0.21	0.57	0.60	0.59	0.59	0.64	0.59	--	0.39	0.14	0.61	--	-0.97	-0.25	0.00	-0.48
Burst Length	0.54	1.00	-0.62	0.36	0.36	0.37	0.41	0.44	0.28	--	0.59	0.36	0.27	--	-0.60	-0.46	0.23	-0.46
Frequency	-0.21	-0.62	1.00	0.07	0.09	0.11	0.02	-0.01	0.22	--	-0.54	-0.28	-0.49	--	0.61	0.15	-0.38	0.44
Reflectance LI	0.57	0.36	0.07	1.00	0.99	0.95	0.88	0.87	0.66	--	-0.26	-0.51	-0.24	--	-0.41	-0.03	0.08	-0.37
Reflectance LII	0.60	0.36	0.09	0.99	1.00	0.97	0.92	0.90	0.67	--	-0.29	-0.53	-0.24	--	-0.41	-0.01	0.09	-0.38
Reflectance LIII	0.59	0.37	0.11	0.95	0.97	1.00	0.98	0.96	0.65	--	-0.35	-0.58	-0.25	--	-0.38	0.03	0.11	-0.39
Reflectance LIV	0.59	0.41	0.02	0.88	0.92	0.98	1.00	0.98	0.55	--	-0.30	-0.55	-0.21	--	-0.40	0.03	0.14	-0.40
Reflectance LV	0.64	0.44	-0.01	0.87	0.90	0.96	0.98	1.00	0.58	--	-0.21	-0.46	-0.08	--	-0.47	0.02	0.24	-0.49

Reflectance LVI	0.59	0.28	0.22	0.66	0.67	0.65	0.55	0.58	1.00	--	-0.37	-0.34	0.66	--	-0.20	0.60	0.99	-0.71
Deformity	--	--	--	--	--	--	--	--	--	--	--	--	--	--	--	--	--	--
Surface Righting	0.39	0.59	-0.54	-0.26	-0.29	-0.35	-0.30	-0.21	-0.37	--	1.00	0.92	0.39	--	-0.45	-0.72	-0.25	0.01
Negative Geotaxis	0.14	0.36	-0.28	-0.51	-0.53	-0.58	-0.55	-0.46	-0.34	--	0.92	1.00	0.42	--	-0.24	-0.72	-0.17	-0.03
Air Righting	0.61	0.27	-0.49	-0.24	-0.24	-0.25	-0.21	-0.08	0.66	--	0.39	0.42	1.00	--	-0.58	-0.03	0.55	-0.66
Auditory Startle	--	--	--	--	--	--	--	--	--	--	--	--	--	--	--	--	--	--
Visual Placing	-0.97	-0.60	0.61	-0.41	-0.41	-0.38	-0.40	-0.47	-0.20	--	-0.45	-0.24	-0.58	--	1.00	0.44	0.08	0.54
Marble Burying	-0.25	-0.46	0.15	-0.03	-0.01	0.03	0.03	0.02	0.60	--	-0.72	-0.72	-0.03	--	0.44	1.00	0.51	0.10
Self-grooming	0.00	0.23	-0.38	0.08	0.09	0.11	0.14	0.24	0.99	--	-0.25	-0.17	0.55	--	0.08	0.51	1.00	-0.71
Social Preference	-0.48	-0.46	0.44	-0.37	-0.38	-0.39	-0.40	-0.49	-0.71	--	0.01	-0.03	-0.66	--	0.54	0.10	-0.71	1.00

**3-month-old VPA weak female (N=9)**	**Lat**	**Bh**	**Freq**	**R LI**	**R LII**	**R LIII**	**R LIV**	**R LV**	**R LVI**	**Def**	**SRR**	**NGR**	**ARR**	**AS**	**VPR**	**MB**	**SG**	**SP**
Latency	1.00	-0.07	0.31	-0.46	-0.44	-0.38	-0.37	-0.41	-0.38	0.09	0.58	-0.09	-0.55	0.03	-0.32	0.23	-0.34	-0.50
Burst Length	-0.07	1.00	-0.17	0.16	0.16	0.15	0.16	0.22	0.33	0.43	0.02	0.16	0.59	-0.08	-0.30	0.24	0.66	-0.43
Frequency	0.31	-0.17	1.00	-0.09	-0.11	-0.10	-0.05	-0.05	0.03	0.27	0.47	-0.45	-0.40	-0.26	-0.19	0.55	0.06	-0.31
Reflectance LI	-0.46	0.16	-0.09	1.00	1.00	0.99	0.99	0.99	0.95	-0.12	-0.53	0.40	0.35	-0.16	0.39	0.27	0.29	0.57
Reflectance LII	-0.44	0.16	-0.11	1.00	1.00	1.00	0.99	0.99	0.95	-0.14	-0.54	0.41	0.34	-0.16	0.39	0.26	0.27	0.58
Reflectance LIII	-0.38	0.15	-0.10	0.99	1.00	1.00	1.00	0.99	0.95	-0.15	-0.52	0.41	0.29	-0.18	0.38	0.30	0.25	0.56
Reflectance LIV	-0.37	0.16	-0.05	0.99	0.99	1.00	1.00	1.00	0.97	-0.13	-0.49	0.38	0.27	-0.21	0.37	0.35	0.26	0.55
Reflectance LV	-0.41	0.22	-0.05	0.99	0.99	0.99	1.00	1.00	0.98	-0.09	-0.49	0.37	0.33	-0.21	0.36	0.35	0.31	0.51
Reflectance LVI	-0.38	0.33	0.03	0.95	0.95	0.95	0.97	0.98	1.00	0.01	-0.39	0.29	0.30	-0.36	0.29	0.51	0.40	0.42
Deformity	0.09	0.43	0.27	-0.12	-0.14	-0.15	-0.13	-0.09	0.01	1.00	0.71	0.15	0.46	0.21	0.32	0.23	0.58	-0.69
Surface Righting	0.58	0.02	0.47	-0.53	-0.54	-0.52	-0.49	-0.49	-0.39	0.71	1.00	0.00	-0.20	0.00	0.00	0.24	0.13	-0.64
Negative Geotaxis	-0.09	0.16	-0.45	0.40	0.41	0.41	0.38	0.37	0.29	0.15	0.00	1.00	0.53	0.40	0.27	-0.32	0.41	0.26
Air Righting	-0.55	0.59	-0.40	0.35	0.34	0.29	0.27	0.33	0.30	0.46	-0.20	0.53	1.00	0.51	0.26	-0.32	0.72	-0.13
Auditory Startle	0.03	-0.08	-0.26	-0.16	-0.16	-0.18	-0.21	-0.21	-0.36	0.21	0.00	0.40	0.51	1.00	0.24	-0.72	0.07	-0.28
Visual Placing	-0.32	-0.30	-0.19	0.39	0.39	0.38	0.37	0.36	0.29	0.32	0.00	0.27	0.26	0.24	1.00	-0.07	-0.05	0.21
Marble Burying	0.23	0.24	0.55	0.27	0.26	0.30	0.35	0.35	0.51	0.23	0.24	-0.32	-0.32	-0.72	-0.07	1.00	0.24	-0.17
Self-grooming	-0.34	0.66	0.06	0.29	0.27	0.25	0.26	0.31	0.40	0.58	0.13	0.41	0.72	0.07	-0.05	0.24	1.00	-0.26
Social Preference	-0.50	-0.43	-0.31	0.57	0.58	0.56	0.55	0.51	0.42	-0.69	-0.64	0.26	-0.13	-0.28	0.21	-0.17	-0.26	1.00

**3-month-old VPA strong female (N=6)**	**Lat**	**Bh**	**Freq**	**R LI**	**R LII**	**R LIII**	**R LIV**	**R LV**	**R LVI**	**Def**	**SRR**	**NGR**	**ARR**	**AS**	**VPR**	**MB**	**SG**	**SP**
Latency	1.00	-0.16	-0.37	0.04	0.03	0.04	0.08	0.11	0.08	-0.44	0.06	-0.39	-0.40	-0.44	-0.44	-0.34	0.00	-0.08
Burst Length	-0.16	1.00	-0.31	0.62	0.61	0.61	0.61	0.60	0.58	0.13	-0.50	0.60	0.80	0.13	-0.30	0.63	-0.41	-0.87
Frequency	-0.37	-0.31	1.00	-0.67	-0.67	-0.66	-0.65	-0.64	-0.61	0.21	0.41	-0.28	0.05	0.21	0.40	-0.70	-0.32	0.47
Reflectance LI	0.04	0.62	-0.67	1.00	1.00	1.00	1.00	0.99	0.99	0.29	-0.48	0.49	0.70	0.29	0.18	0.70	0.00	-0.38
Reflectance LII	0.03	0.61	-0.67	1.00	1.00	1.00	0.99	0.99	0.99	0.29	-0.49	0.49	0.70	0.29	0.18	0.71	0.02	-0.38
Reflectance LIII	0.04	0.61	-0.66	1.00	1.00	1.00	1.00	0.99	0.99	0.29	-0.49	0.48	0.70	0.29	0.19	0.69	0.01	-0.38
Reflectance LIV	0.08	0.61	-0.65	1.00	0.99	1.00	1.00	1.00	1.00	0.29	-0.47	0.45	0.71	0.29	0.19	0.65	-0.01	-0.37
Reflectance LV	0.11	0.60	-0.64	0.99	0.99	0.99	1.00	1.00	1.00	0.29	-0.45	0.44	0.71	0.29	0.20	0.61	-0.04	-0.35
Reflectance LVI	0.08	0.58	-0.61	0.99	0.99	0.99	1.00	1.00	1.00	0.29	-0.47	0.42	0.71	0.29	0.22	0.62	-0.01	-0.33
Deformity	-0.44	0.13	0.21	0.29	0.29	0.29	0.29	0.29	0.29	1.00	0.55	0.77	0.54	1.00	0.79	-0.03	-0.60	0.33
Surface Righting	0.06	-0.50	0.41	-0.48	-0.49	-0.49	-0.47	-0.45	-0.47	0.55	1.00	0.19	-0.30	0.55	0.43	-0.74	-0.55	0.64
Negative Geotaxis	-0.39	0.60	-0.28	0.49	0.49	0.48	0.45	0.44	0.42	0.77	0.19	1.00	0.64	0.77	0.28	0.43	-0.62	-0.28

Air Righting	-0.40	0.80	0.05	0.70	0.70	0.70	0.71	0.71	0.71	0.54	-0.30	0.64	1.00	0.54	0.30	0.47	-0.44	-0.42
Auditory Startle	-0.44	0.13	0.21	0.29	0.29	0.29	0.29	0.29	0.29	1.00	0.55	0.77	0.54	1.00	0.79	-0.03	-0.60	0.33
Visual Placing	-0.44	-0.30	0.40	0.18	0.18	0.19	0.19	0.20	0.22	0.79	0.43	0.28	0.30	0.79	1.00	-0.17	-0.11	0.72
Marble Burying	-0.34	0.63	-0.70	0.70	0.71	0.69	0.65	0.61	0.62	-0.03	-0.74	0.43	0.47	-0.03	-0.17	1.00	0.31	-0.61
Self-grooming	0.00	-0.41	-0.32	0.00	0.02	0.01	-0.01	-0.04	-0.01	-0.60	-0.55	-0.62	-0.44	-0.60	-0.11	0.31	1.00	0.16
Social Preference	-0.08	-0.87	0.47	-0.38	-0.38	-0.38	-0.37	-0.35	-0.33	0.33	0.64	-0.28	-0.42	0.33	0.72	-0.61	0.16	1.00
